# Blocking VCAM-1 ameliorates hypertensive cardiac remodeling by impeding macrophage infiltration

**DOI:** 10.3389/fphar.2022.1058268

**Published:** 2022-11-17

**Authors:** Ze-Yang Qiu, Wei-Jia Yu, Jie Bai, Qiu-Yue Lin

**Affiliations:** Institute of Cardiovascular Diseases, First Affiliated Hospital of Dalian Medical University, Dalian, China

**Keywords:** VCAM-1, hypertension, macrophage infiltration, inflammation, cardiac remodeling

## Abstract

Cardiac remodeling is an important mechanism of heart failure, which frequently results from leukocyte infiltration. Vascular cellular adhesion molecule-1 (VCAM-1) plays a critical role in leukocyte adhesion and transmigration. However, the importance of VCAM-1 in the development of angiotensin II (Ang II)-induced cardiac remodeling remains unclear. Wild-type (WT) mice were infused with Ang II (1,000 ng/kg/min) for 14 days and simultaneously treated with VCAM-1 neutralizing antibody (0.1 or 0.2 mg) or IgG control. Systolic blood pressure (SBP) and cardiac function were detected by a tail-cuff and echocardiography. Cardiac remodeling was evaluated by histological staining. Adhesion and migration of bone marrow macrophages (BMMs) were evaluated *in vitro*. Our results indicated that VCAM-1 levels were increased in the serum of patients with heart failure (HF) and the hearts of Ang II-infused mice. Furthermore, Ang II-caused hypertension, cardiac dysfunction, hypertrophy, fibrosis, infiltration of VLA-4+ BMMs and oxidative stress were dose-dependently attenuated in mice administered VCAM-1 neutralizing antibody. In addition, blocking VCAM-1 markedly alleviated Ang II-induced BMMs adhesion and migration, therefore inhibited cardiomyocyte hypertrophy and fibroblast activation. In conclusion, the data reveal that blocking VCAM-1 ameliorates hypertensive cardiac remodeling by impeding VLA-4+ macrophage infiltration. Selective blockage of VCAM-1 may be a novel therapeutic strategy for hypertensive cardiac diseases.

## Introduction

Hypertension can lead to numerous cardiovascular diseases (CVDs), especially hypertensive heart disease (HHD) (Dorans et al., 2018). Cardiac hypertrophy or dysfunction is the main features of HHD, which frequently results in symptomatic heart failure (HF) (Frohlich et al., 1992). In general, it is believed that in HHD, elevated systemic blood pressure causes cardiac afterload to increase, and the LV undergoes extensive growth to resist the pressure overload and maintain normal cardiac output, resulting in LV hypertrophy (Izzo and Gradman, 2004; Díez et al., 2005; Drazner, 2011). Pathological cardiac remodeling is one of the typical functional and structural changes that occur in response to cardiovascular damage. This process involves several cellular changes, including cardiomyocyte hypertrophy and apoptosis, myofibroblast activation and secretion of fibrillar collagen, which ultimately lead to HF (Shimizu and Minamino, 2016; Wu et al., 2017).

Immune cells, including macrophages and lymphocytes, can release a wide range of cytokines, chemokines and adhesion molecules under inflammatory conditions, which play a significant role in pathological cardiac remodeling (Taube et al., 2012; Kong et al., 2018). Vascular cellular adhesion molecule-1 (VCAM-1) is a 90-kDa glycoprotein that mainly expressed in endothelial cells. VCAM-1 expression is upregulated by TNF-α, ROS, oxidized LDL, and TLR agonists through NF-κB and AP1 signaling (Cook-Mills et al., 2011). Experimental data have indicated that VCAM-1 contributes to adhesion and migration of leukocytes by interacting with the integrin receptor α4β1 (VLA-4) (Troncoso et al., 2021). Clinical investigations have also indicated that VCAM-1 level is elevated in patients with ischemic cardiomyopathy (Theiss et al., 2007). Increasing evidence shows that VCAM-1 is closely related with the development of CVDs, such as ischemic cardiomyopathy, hypertension, coronary disease and atrial fibrillation (Yin et al., 2022). Moreover, our recent experimental data confirmed that VCAM-1 level is highly elevated in Ang II-infused aorta, which promotes monocyte migration and adhesion to endothelial, the subsequent differentiation of these cells into macrophages and the release of proinflammatory mediators, resulting in hypertension and vascular dysfunction (Yin et al., 2022). However, the significance of VCAM-1 in the progression of hypertensive myocardial remodeling remains unclear.

In our current investigation, by administering a VCAM-1 neutralizing antibody *in vivo* and *in vitro* coculture system, we explored the effect of VCAM-1 on macrophage adhesion and migration as well as cardiac remodeling and dysfunction induced by Ang II. Our data confirmed that VCAM-1 expressions were elevated in both clinical HF patients and Ang II-treated mouse heart. Furthermore, blocking VCAM-1 obviously attenuated Ang II-caused arterial hypertension, infiltration of VLA-4-positive macrophages, and expression of pro-inflammatory cytokines (IL-1β, IL6 and TNF-ɑ) and ROS production, which activating multiple signaling pathways leading to amelioration of cardiac remodeling and dysfunction. Together, our evidence shows novel viewpoint that blocking VCAM-1 exerts a cardioprotective effect against Ang II treatment and that targeting VCAM-1 may represent a new therapeutic strategy for hypertensive cardiac diseases.

## Materials and methods

### Animals

All experimental protocols were approved by the Institutional Animal Care and Use Committee (IACUC) of the First Affiliated Hospital of Dalian Medical University (AEE20027). The wild-type (WT) male C57BL/6J mice were fed under specific pathogen-free conditions for 8–10 weeks. Mice were anesthesia with 1.5% isoflurane, then the osmotic pumps (Alzet, 1,002; DURECT, Cupertino, CA, United States) were subcutaneously implanted to deliver angiotensin (Ang) II (1,000 ng kg^−1^ min^−1^) for 14 days to construct a hypertensive cardiac remodeling model. Then anti-VCAM-1 neutralizing antibody (0.1 or 0.2 mg, BE0027, Bioxcell) or control IgG was intraperitoneally (i.p.) injected every 2 days as previously described (Yin et al., 2022).

### Systolic blood pressure and cardiac function

The mouse systolic blood pressure (SBP) of each group was measured and recorded with a tail-cuff instrument (BP-98A, Softron, Japan). All mice were placed in a restraint device on a warming platform to keep calm for at least half an hour. Then the SBP was measured with a tail cuff after mouse heart rate was stabilized as previously described (Wang et al., 2016). The mouse cardiac function was measured by echocardiography after Ang II infusion for 14 days as previously described (Wang et al., 2018). Briefly, all mice were anesthesia with 1.5% isoflurane and keep heart rate steady, then placed on a warming platform. The M-model echocardiographic analysis was performed by a 30 MHz probe (Vevo 1,100 system; VisualSonics, Toronto, Ontario, Canada). The parameters of ejection fraction, fractional shortening, ventricular wall and internal dimension at end-diastole and end-systole were captured and calculated.

### Histopathology

All mice were anesthesia with an overdose of isoflurane (>5%), and hearts were carefully collected. The hearts were fix by 4% paraformaldehyde and dehydrated, and then embedded in paraffin or OCT. Cardiac paraffin sections (4 μm) were performed hematoxylin-eosin (HE) staining, Masson’s trichrome staining and immunohistochemical staining with antibodies against VCAM-1 (1:200, Proteintech), α-smooth muscle actin (α-SMA, 1:200, Arigo), CD68 (1:200, Abcam) and nitrotryrosine (1:200, Bioss) as previously described (Wang et al., 2018). Eight-micrometer frozen sections were subjected to wheat germ agglutinin (WGA) staining, dihydroethidium (DHE) staining and immunofluorescence staining with antibodies against collagen III (1:200, Abcam), VLA-4 (1:200, Bioss) and γ-H2AX (a sensitive marker of DNA damage, 1:200, Abcam) as previously described (Wang et al., 2018; Yin et al., 2022).

### Quantitative polymerase chain reaction

Total RNA was extracted from heart tissues by Trizol regent (Sangon Biotech, B511311) according to the manufacturer’s instructions. Total RNA (1–2 μg) was used to synthesize the single stranded cDNA by using the PrimeScript RT Kit (Yeasen, 11141ES60). Quantitative polymerase chain reaction (qPCR) was performed with an Applied Biosystems 7,500 system after the cDNA samples were mixed with SYBR reagent (Accurate Biotechnology Co., Ltd., Hunan, AG11701). The specific primer sequences of VCAM-1, ANF, BNP, MYH7, collagen I, collagen III, α-SMA, IL-1β, IL-6, TNF-α, NOX1, NOX2, NOX4, GAPDH and β-actin are listed in Table 1.

**TABLE 1:** Primers used for quantitative real-time PCR analysis.

Gene	Forward primer (5′-3′)	Reverse primer (5′-3′)
VCAM-1	GTT​CCA​GCG​AGG​GTC​TAC​C	AAC​TCT​TGG​CAA​ACA​TTA​GGT​GT
ANF	CAC​AGA​TCT​GAT​GGA​TTT​CAA​GA	CCT​CAT​CTT​CTA​CCG​GCA​TC
BNP	GAA​GGT​GCT​GTC​CCA​GAT​GA	CCA​GCA​GCT​GCA​TCT​TGA​AT
MYH7	GCG​AGA​GTG​AAC​AGC​AAG​AGT	ATG​GCT​GAG​CCT​TGG​ATT​CT
Collagen I	GAG​TAC​TGG​ATC​GAC​CCT​AAC​CA	GAC​GGC​TGA​GTA​GGG​AAC​ACA
Collagen III	TCC​CCT​GGA​ATC​TGT​GAA​TC	TGA​GTC​GAA​TTG​GGG​AGA​AT
α-SMA	TCC​TGA​CGC​TGA​AGT​ATC​CGA​TA	GGC​CAC​ACG​AAG​CTC​GTT​AT
IL-1β	TGC​CAC​CTT​TTG​ACA​GTG​ATG	TGA​TGT​GCT​GCT​GCG​AGA​TT
IL-6	TGA​TGG​ATG​CTA​CCA​AAC​TGG​A	TGT​GAC​TCC​AGC​TTA​TCT​CTT​GG
TNF-α	CAG​GCG​GTG​CCT​ATG​TCT​C	CGA​TCA​CCC​CGA​AGT​TCA​GTA​G
NOX1	CCT​GAT​TCC​TGT​GTG​TCG​AAA	TTG​GCT​TCT​TCT​GTA​GCG​TTC
NOX2	CTT​CTT​GGG​TCA​GCA​CTG​GC	GCA​GCA​AGA​TCA​GCA​TGC​AG
NOX4	CTT​GGT​GAA​TGC​CCT​CAA​CT	TTC​TGG​GAT​CCT​CAT​TCT​GG
GAPDH	GGT​TGT​CTC​CTG​CGA​CTT​CA	GGT​GGT​CCA​GGG​TTT​CTT​ACT​C
β-actin	GTG​ACG​TTG​ACA​TCC​GTA​AAG​A	GCC​GGA​CTC​ATC​GTA​CTC​C

VCAM-1, vascular cell adhesion molecule one; ANF, atrial natriuretic factor; BNP, brain natriuretic factor; MYH7, myosin heavy chain seven; α-SMA, α-smooth muscle actin; IL-1β, interleukin one beta; IL-6, interleukin six; TNF-α, tumor necrosis factor alpha; NOX1, NADPH, oxidase one; NOX2, NADPH, oxidase two; NOX4, NADPH, oxidase four; GAPDH, glyceraldehyde 3-phosphate dehydrogenase; β-actin, beta Cytoskeletal Actin.

### Immunoblotting

Protein was isolated from cardiac tissue or cells by an extraction kit (Keygenbio, KGP250). Protein concentration was calculated by using a BCA Protein Assay Kit (Life-iLab, AP12L025). Samples (30–50 μg) were separated on SDS-polyacrylamide gels and transferred onto PVDF membranes. The membranes were incubated with appropriate antibodies against VCAM-1 (1:200, Proteintech), calcineurin A (1:800, CST), phosphor (p)-STAT3 (1:500, CST), STAT3 (1:500, Arigo), TGF-β1 (1:800, CST), p-Smad2/3 (1:500, CST), Smad2/3 (1:1,000, CST), p-IKKα (1:500, CST), IKKα (1:1,000, CST), p-p65 (1:500, CST), p65 (1:1,000, CST), NOX1 (1:800, CST), NOX4 (1:800, CST) and GAPDH (1:2000, HUABIO). Then, membranes were incubated with horseradish peroxidase-conjugated secondary antibodies (Sino Biological Inc.). Blots were visualized using an ECL chemiluminescence system. GADPH served as the internal reference.

### Human blood sample

Human serum was collected from the First Affiliated Hospital of Dalian Medical University and Beijing Chao-Yang Hospital of Capital Medical University between August 2021 and January 2022. The criteria of HF diagnosis and treatment were selected according to the 2021 ESC Guidelines (McDonagh et al., 2021), and 30 HF patients and 30 health control were included in the current study. All patients and controls underwent routine physical examinations, echocardiography, urine and blood routine tests. The functional examinations of the lever, lung, kidney and lung were conducted. HF patients with a reduced LV ejection fraction (LVEF <40%), increased BNP concentration (BNP >35 pg/ml) as well as clinical symptoms and signs of HF within 6 months are included. The patients with unstable angina or myocardial infarction during the last 6 months, autoimmune or infectious diseases, cancer, lung disease, and renal failure were excluded. The control subjects with normal cardiac function and without any cardiovascular risk factors, cardiac diseases or any family history of coronary artery disease. as previously described (Wang et al., 2018). The experimental process was approved by the Ethics Committees of First Affiliated Hospital of Dalian Medical University (No. LCKY 2016-31) and Beijing Chao-Yang Hospital of Capital Medical University (2022-human-244). VCAM-1 concentration was measured by ELISA according to the manufacturer’s instructions (Elabscience).

### Macrophage adhesion and migration

Mouse bone marrow macrophages (BMMs) was isolated and cultured in medium containing 10% fetal bovine serum (Life-iLab, AC03L055) as previously described (Yin et al., 2022). Briefly, the tibia and femur from WT mice were isolated, the whole BM cells were flushed out and cultured overnight. The suspended blood cells were removed and the adherent BM cells were stimulated by recombinant murine colony stimulating factor (M-CSF, PeproTeCH) to induce macrophage maturation. The VLA-4^+^ macrophages were confirmed by immunostaining with anti-VLA-4 antibody. Human umbilical vein endothelial cells (HUVECs) were cultured in EC medium and pretreated with anti-VCAM-1 (5 μg/ml) or IgG control for 4 h and then stimulated with Ang II (100 nM) or saline for an additional 24 h as describe previously (Yin et al., 2022). For the adhesion assay, BMMs were fluorescently labeled with PKH26 (Red, Sigma‒Aldrich) and added to the HUVECs that had been treated as described above. For the migration experiment, BMMs were seeded in the upper chamber of a Transwell insert, which was inserted into plates containing HUVECs treated as described above. DAPI staining was performed to identify migrated BMMs. Images were obtained by microscopy (Olympus, IX73, 100 x), and six randomly selected fields were analyzed.

### Coculture of BMMs and primary cardiomyocytes or fibroblasts

Neonatal rat Primary cardiomyocytes (NRCMs) and cardiac fibroblasts (CFs) were obtained enzymatically from 1-day-old Sprague-Dawley rat hearts as previously described (Wang et al., 2018). Briefly, hearts were digested into single-cell suspensions and cultured in DMEM/F-12 medium for 90 min. The adherent fibroblasts were cultured in DMEM medium (VivaCell, Shanghai, China, C3113-0500), and the suspended cardiomyocytes was harvested and cultured in new DMEM/F-12 medium, which containing 5-Brdu (100 μm) to eliminate residual CFs. A coculture system was constructed with a transwell plate as previously described (Wang et al., 2018). BMMs and HUVECs were co-cultured and treated with anti-VCAM-1 (5 μg/ml) and Ang II (100 nM) as described in Macrophage Adhesion and Migration. The supernatants were collected and added to CMs or CFs for 24 h. Cardiomyocyte size was determined using immunostaining with anti-α-actinin antibody. The protein levels of CaNA, STAT3, TGF-β1 and p-Smad2/3 in lysates from CMs or CFs were evaluated by immunoblotting analysis. The mRNA levels of collagen I and collagen III in CFs were assessed by qPCR analysis.

### Statistical analysis

All data are expressed as the mean ± standard deviation (M ± SD) and statistically analyzed using SPSS 19.0. Data distribution and heteroscedasticity were analyzed by the Shapiro–Wilk test. Independent *t* test, one-way ANOVA, or chi-square test were utilized to compare differences in data. *P* < 0.05 was considered statistically significant.

## Results

### VCAM-1 level is enhanced in HF patients and Ang II-infused mouse heart

**FIGURE 1:**
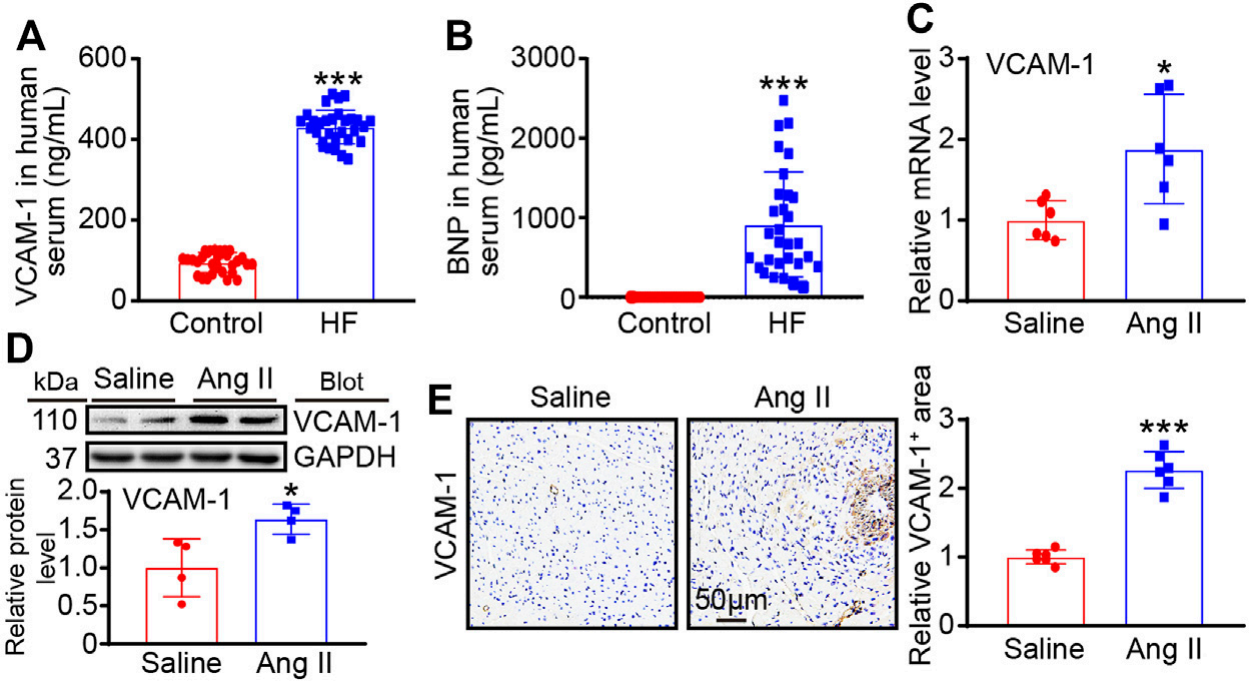
VCAM-1 is upregulated in HF patients and Ang II-infused mice. **(A)** Analysis of serum VCAM-1 levels in HF patients and control subjects by ELISA (*n* = 30) **(B)** Analysis of serum BNP levels in HF patients and control subjects by ELISA (*n* = 30) **(C)** WT mice were infused with saline or Ang II (1,000 ng/kg/min) for 14 days qPCR analysis of VCAM-1 mRNA levels in heart tissues (*n* = 6) **(D)** Immunoblot analysis of VCAM-1 protein levels in heart tissues (top) and quantification of relative protein levels (bottom, *n* = 4) **(E)** Immunohistochemical staining of VCAM-1 in heart sections (left). Scale bar: 50 μm. Quantification of the VCAM-1-positive area (right, *n* = 6). The data are expressed as the M ± SD, and n represents the number of human subjects or animal samples. *p < 0.05 vs saline group and ***p < 0.001 vs saline group or control group.

To verify VCAM-1 participation in Ang II-induced hypertensive cardiac remodeling and clinical HF, we first measured the serum VCAM-1 level in HF patients. The baseline characteristics of humans are shown in Table 2. Serum VCAM-1 and BNP levels were significantly upregulated in HF patients compared with normal controls (Figures 1A,B). Moreover, we measured VCAM-1 level in mouse heart after Ang II treatment. qPCR indicated that VCAM-1 mRNA level was markedly increased in Ang II-infused mouse hearts (Figure 1C). The increase in the expression of VCAM-1 in the hearts of Ang II-infused mice was verified by both immunoblotting and immunohistochemical analysis (Figure 1D,E). Therefore, these results show that VCAM-1 upregulation may be associated with the development of Ang II-induced cardiac remodeling.

**TABLE 2:** Baseline characteristics of patients with heart failure (HF) or control subjects.

Parameters	Control (n = 30)	HF (n = 30)	*p*-Value
Male, n (%)	33.33	63.33	0.020
Age, years	58.17 ± 5.33	67.63 ± 13.49	0.000
LVEF, %	59.93 ± 0.36	36.07 ± 7.00	0.000
BNP, pg/mL	0.22 ± 0.04	915.57 ± 648.99	0.000
SBP, mmHg	119.20 ± 10.42	135.03 ± 26.55	0.012
DBP, mmHg	73.13 ± 8.49	78.00 ± 18.31	0.201
Heart rate, bpm	66.77 ± 7.14	77.27 ± 17.42	0.006
Total cholesterol, mmol/L	5.45 ± 0.94	4.18 ± 1.06	0.000
HDL, mmol/L	1.38 ± 0.33	0.95 ± 0.24	0.000
LDL, mmol/L	3.02 ± 0.64	2.34 ± 0.77	0.000
Triglycerides, mmol/L	1.73 ± 0.89	1.67 ± 1.04	0.375
Blood glucose, mmol/L	5.23 ± 0.64	6.05 ± 2.35	0.145
Leucocyte, 10^9^/L	5.40 ± 1.21	6.71 ± 2.85	0.065
Creatinine, μmol/L	61.83 ± 11.62	120.13 ± 84.03	0.000

Values: means ± SD.

*p* < 0.05 was considered statistically significant.

LVEF, left ventricular ejective fraction; BNP, brain natriuretic factor; HDL, high density lipoprotein; LDL, low density lipoprotein; SBP, systolic blood pressure; DBP, diastolic blood pressure.

### Inhibition of VCAM-1 attenuates Ang II-caused hypertension, cardiac hypertrophy and cardiac dysfunction

**FIGURE 2:**
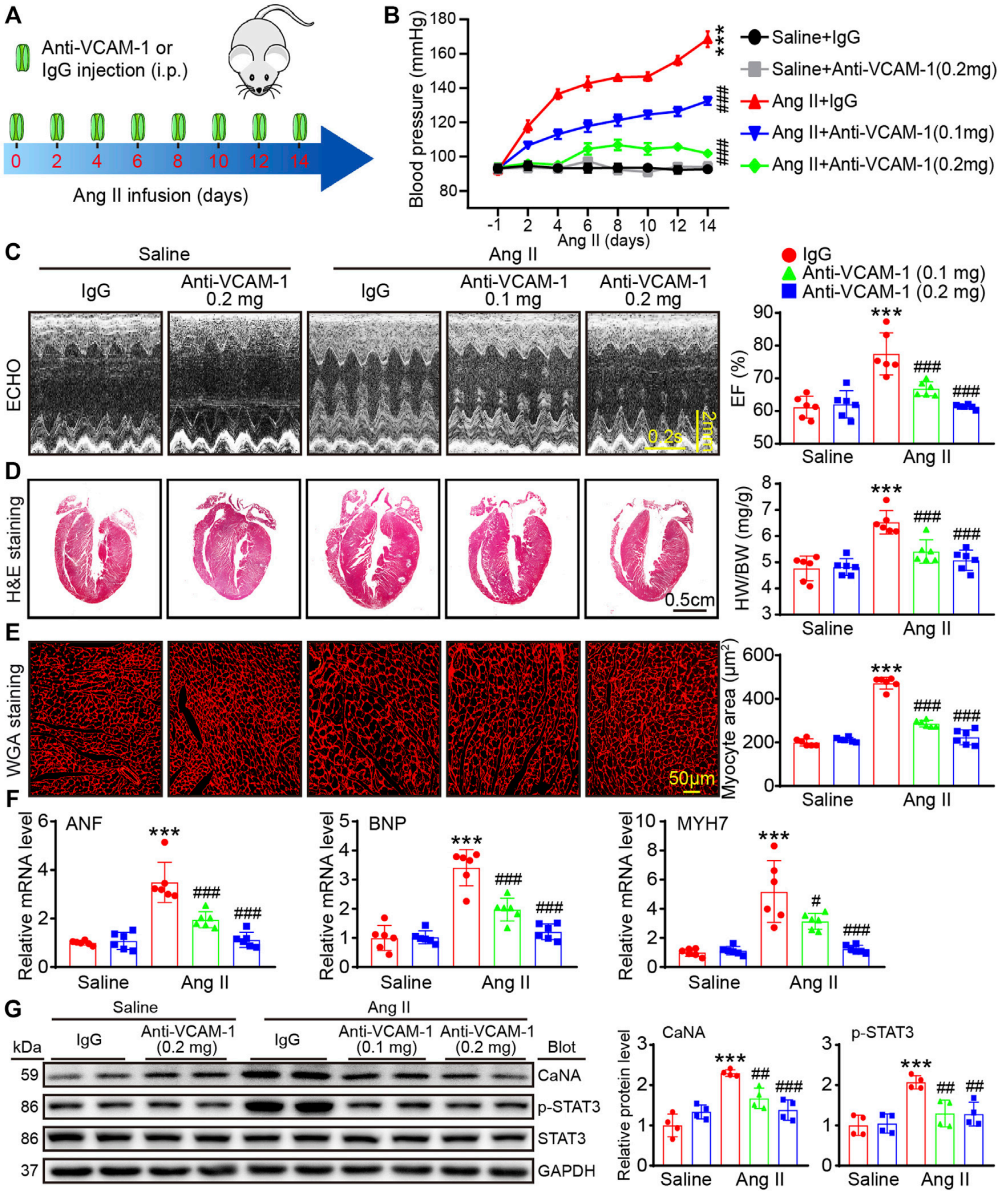
Inhibition of VCAM-1 attenuates Ang II-induced hypertension, cardiac hypertrophy and cardiac dysfunction. **(A)** WT mice were infused with saline or Ang II (1,000 ng/kg/min) for 14 days and cotreated with a VCAM-1 neutralizing antibody at a dose of 0.1 and 0.2 mg/mouse or IgG control every 2 days **(B)** Systolic blood pressure (SBP) in each group (*n* = 6) **(C)** Echocardiographic analysis (left) and quantification of the ejection fraction (EF%) (right, *n* = 6) **(D)** H&E staining of heart sections (left). Scale bar: 0.5 cm. The ratio of heart weight to body weight (HW/BW) (right, *n* = 6) **(E)** WGA staining of heart sections (left). Scale bar: 50 μm. Analysis of the cross-sectional area of myocytes (right, *n* = 6) **(F)** qPCR analysis of ANF, BNP and MYH7 levels in the heart (*n* = 6) **(G)** Immunoblot analysis of cardiac calcineurin A (CaNA) and p-STAT3 (left) and analysis of the level of these proteins (right, *n* = 4). The data are expressed as the M ± SD, and n represents the number of animals. ***p < 0.001 vs saline + IgG group. ^#^ *p* < 0.05, ^##^ *p* < 0.01 and ^###^ *p* < 0.001 vs Ang II + IgG group.

To determine the role of VCAM-1 in cardiac remodeling, WT mice were treated with a VCAM-1 neutralizing antibody (0.1 or 0.2 mg/mouse) or IgG every 2 days and infused with Ang II (1,000 ng/kg/min) for 14 days (Figure 2A). We found that compared with saline, Ang II observably elevated the mouse systolic blood pressure (SBP), whereas anti-VCAM-1 significantly inhibited this increase in a dose-dependent manner (Figure 2B). The echocardiography data showed that Ang II-induced cardiac dysfunction, as reflected by increased EF% and FS%, was dose-dependently alleviated by anti-VCAM-1 (Figure 2C; Table 3). Next, we analyzed the inhibited effect of VCAM-1 inhibition on cardiac hypertrophy. H&E and WGA staining indicated that Ang II promoted cardiac hypertrophy, as indicated by increases in heart size, the heart weight to body weight (HW/BW) ratio, the cross-sectional area of myocytes, and the mRNA levels of ANF, BNP and MYH7, while these effects were dose-dependently inhibited by anti-VCAM-1 (Figures 2D–F). Accordingly, we examined the hypertrophy-related signaling molecules in mouse heart. Immunoblotting showed that compared with IgG, anti-VCAM-1 treatment dose-dependently blocked Ang II-induced increase in calcineurin A (CaNA) and phosphorylated (p)-STAT3 protein levels (Figure 2G). Together, these data show that VCAM-1 overexpression accelerates pathological cardiac hypertrophy.

**TABLE 3:** Echocardiographic parameters of WT mice treated with Anti-VCAM-1 antibody or IgG control after Ang II infusion.

Parameter	Saline		Ang II		
	IgG	Anti-VCAM-1 (0.2 mg)	IgG	Anti-VCAM-1 (0.1 mg)	Anti- VCAM-1 (0.2 mg)
EF %	61.18 ± 2.94	62.05 ± 12.96	77.47 ± 17.91^***^	66.80 ± 16.61^###^	61.35 ± 16.34^###^
FS %	32.11 ± 2.04	30.84 ± 6.50	45.31 ± 11.61^***^	35.87 ± 9.86^###^	32.37 ± 9.52^###^
LVAW; d	0.72 ± 0.09	0.71 ± 0.18	1.19 ± 0.33^***^	0.97 ± 0.29^#^	0.85 ± 0.27^###^
LVAW; s	1.11 ± 0.18	1.02 ± 0.27	1.84 ± 0.51^***^	1.45 ± 0.44^#^	1.22 ± 0.41^###^
LVID; d	3.63 ± 0.06	3.48 ± 0.76	3.06 ± 0.79^***^	3.23 ± 0.80	3.87 ± 0.90^###^
LVID; s	2.41 ± 0.14	2.48 ± 0.50	1.85 ± 0.56^***^	2.03 ± 0.55	2.62 ± 0.62^###^
LVPW; d	0.74 ± 0.12	0.69 ± 0.18	1.06 ± 0.27^***^	1.03 ± 0.27	0.71 ± 0.25^###^
LVPW; s	1.07 ± 0.14	1.00 ± 0.22	1.53 ± 0.38^***^	1.47 ± 0.38	0.96 ± 0.36^###^

Values: means ± SD (n = 6).

***p < 0.001 vs Saline + IgG.

^#^ *p* < 0.05 and.

^###^ *p* < 0.001 vs Ang II + IgG.

EF, ejection fraction; FS, fractional shortening; LVAW; d, left ventricular anterior wall at end-diastole; LVAW; s, left ventricular anterior wall at end-systole; LVID; d, left ventricular internal dimension at end-diastole; LVID; s, left ventricular internal dimension at end-systole; LVPW; d, left ventricular posterior wall at end-diastole; LVPW; s, left ventricular posterior wall at end-systole.

### Blockade of VCAM-1 suppresses Ang II-induced myocardial fibrosis

**FIGURE 3:**
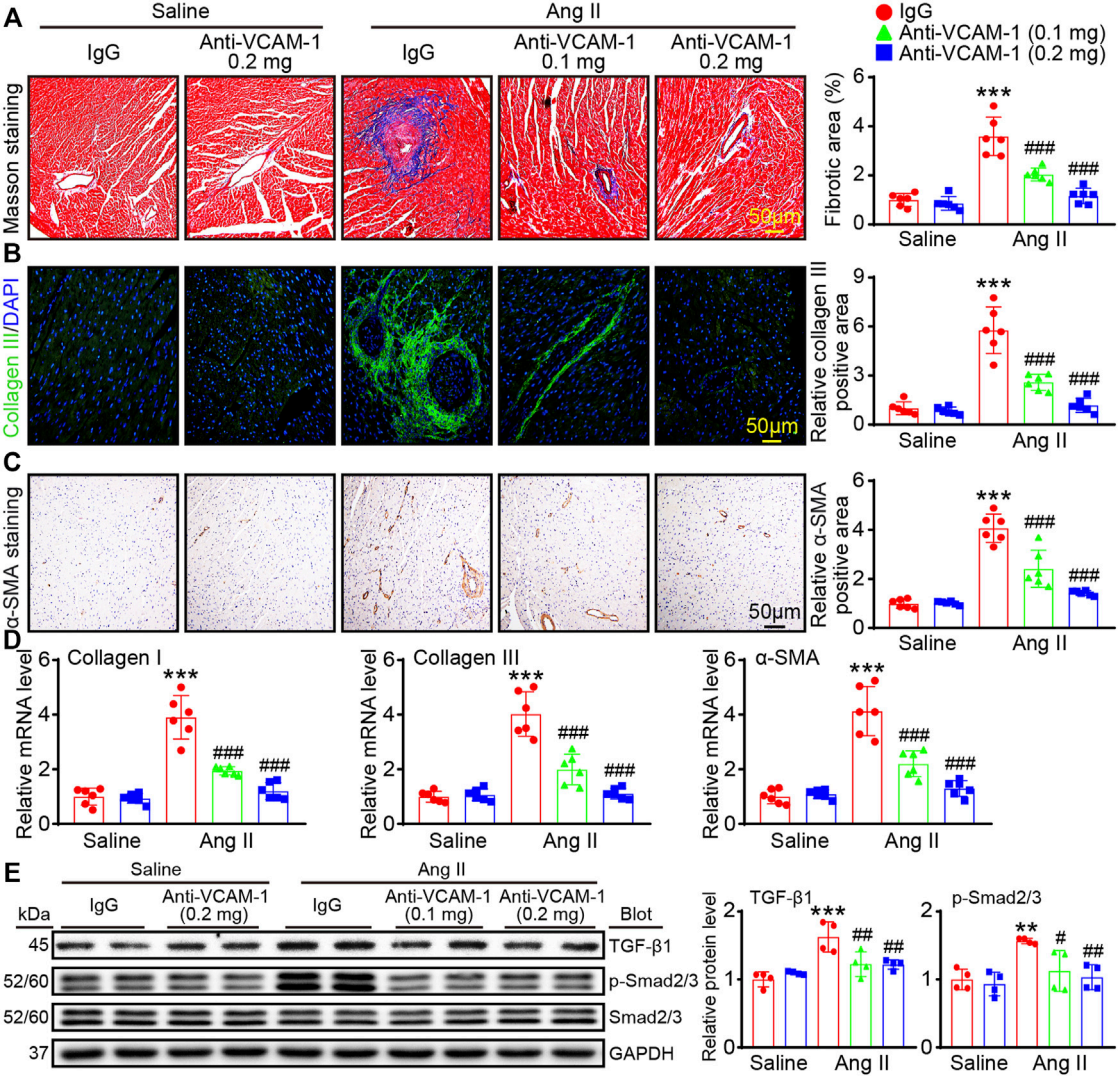
Blockade of VCAM-1 suppresses Ang II induced cardiac fibrosis. **(A)** Masson’s trichrome staining of heart sections (left). Scale bar: 50 μm. Analysis of the fibrotic areas (right, *n* = 6) **(B)** Immunofluorescence staining of heart sections with an anti-collagen III antibody (left). Scale bar: 50 μm. Analysis of collagen III positive areas (right, *n* = 6) **(C)** Immunohistochemical staining of heart sections with an anti-α-SMA antibody (left). Scale bar: 50 μm. Analysis of α-SMA-positive areas (right, *n* = 6) **(D)** qPCR analysis of collagen I, collagen III and α-SMA expression in the heart (*n* = 6) **(E)** Immunoblot analysis of TGF-β1 and p-Smad2/3 (left) and analysis of the levels of these proteins (right, *n* = 4). The data are expressed as the M ± SD, and n represents the number of animals. **p < 0.01 and ***p < 0.001 vs saline + IgG group. ^#^ *p* < 0.05, ^##^ *p* < 0.01 and ^###^ *p* < 0.001 vs Ang II + IgG group.

Cardiac fibrosis often leads to a hallmark of a majority of cardiac diseases. Therefore, we first evaluated the degree of cardiac collagen deposition. Compared with that in the saline group, the fibrotic area in Ang II-infused heart was significantly increase, whereas anti-VCAM-1 antibody dose-dependently alleviated this increase (Figure 3A). Moreover, staining with an anti-collagen III or anti-α-SMA antibody revealed that administration of the anti-VCAM-1 markedly inhibited the Ang II-induced upregulation of collagen III and α-SMA (a marker of myofibroblast activation) in a dose-dependent manner (Figures 3B,C). Similarly, the mRNA expression of collagen I, collagen III and α-SMA were dose-dependently downregulated in anti-VCAM-1 antibody-treated heart compared with IgG-treated heart (Figure 3D). Accordingly, Ang II-induced activation of profibrotic TGF-β1 and p-Smad2/3 in IgG-treated mice was dose-dependently attenuated by anti-VCAM-1 (Figure 3E). Overall, our results indicate that VCAM-1 overexpression promotes cardiac fibrosis.

### Blocking VCAM-1 suppresses Ang II-induced VLA-4^+^ macrophage accumulation and the expression of proinflammatory cytokines

**FIGURE 4:**
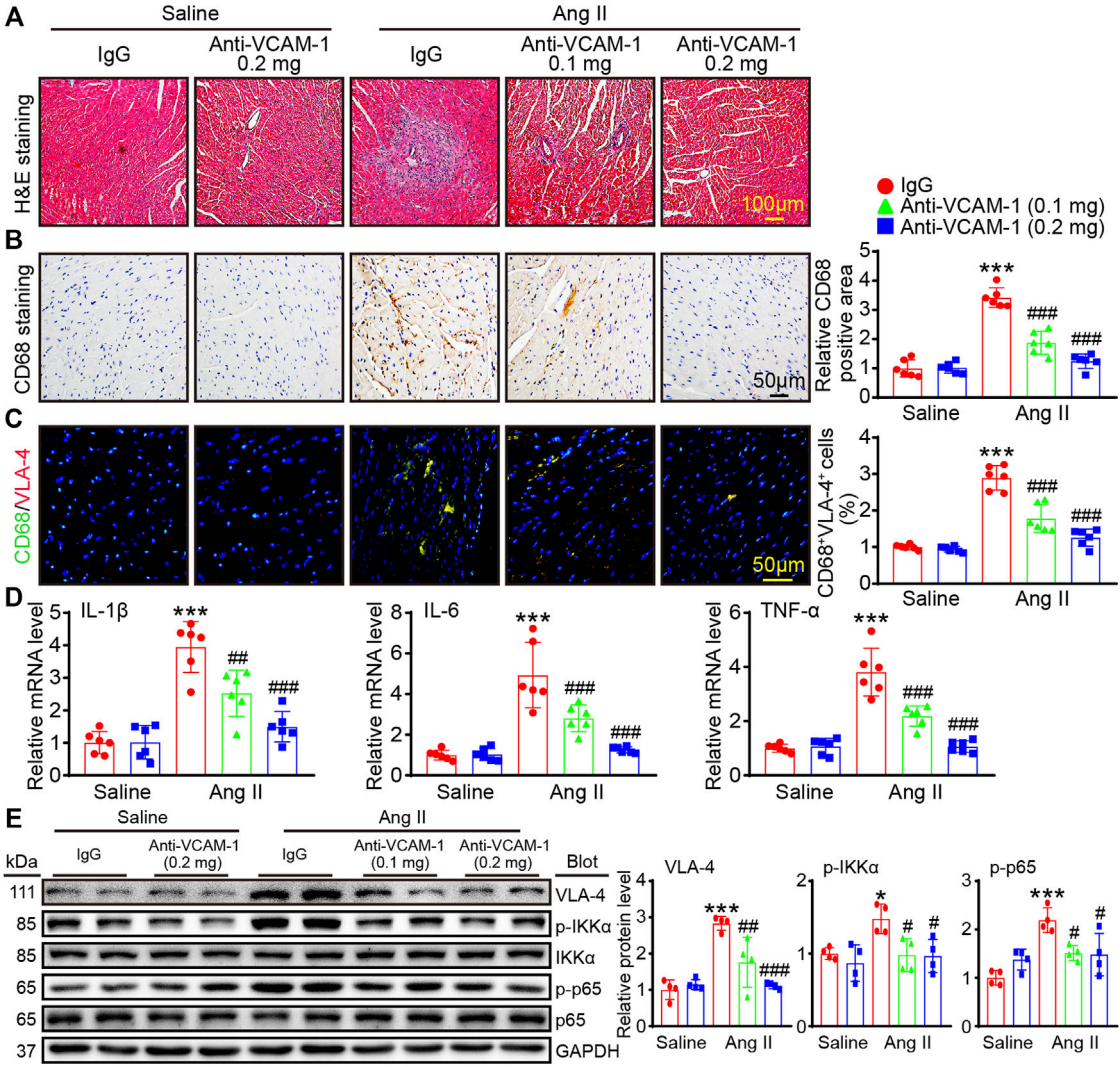
Blocking VCAM-1 inhibits Ang II-induced VLA-4^+^ macrophage infiltration and the expression of proinflammatory cytokines. **(A)** H&E staining of heart sections. Scale bar: 100 μm **(B)** Immunohistochemical staining of heart sections with an anti-CD68 antibody (left). Scale bar: 50 μm. Analysis of CD68-positive macrophages (right, *n* = 6) **(C)** Immunofluorescence staining of heart sections with an anti-CD68 and anti-VLA-4 antibody (left). Scale bar: 50 μm. Analysis of CD68 and VLA-4-positive areas (right, *n* = 6) **(D)** qPCR analysis of IL-1β, IL-6 and TNF-α expression in the heart (*n* = 6) **(E)** Immunoblot analysis of VLA-4, p-IKKα and p-p65 (left) and analysis of the levels of these proteins (right, *n* = 4). The data are expressed as the M ± SD, and n represents the number of animals. *p < 0.05 and ***p < 0.001 vs saline + IgG group. ^#^ *p* < 0.05 and ^###^ *p* < 0.001 vs Ang II + IgG group.

Inflammatory cell infiltration is associated with pathological cardiac remodeling. We next evaluated the role of VCAM-1 in macrophage accumulation and inflammatory reactions. H&E staining revealed that compared with saline control, Ang II infusion promoted cardiac inflammatory cell infiltration, and this effect was dose-dependently inhibited by anti-VCAM-1 treatment (Figure 4A). Then, we identified the infiltrated inflammatory cells using immunohistochemical and immunofluorescence staining with an anti-CD68 and anti-VLA-4 antibody. The results revealed that compared with saline treatment, Ang II infusion observably increased cardiac infiltration of CD68^+^ and VLA-4^+^ macrophages, and this effect was dose-dependently attenuated in anti-VCAM-1-treated group (Figures 4B,C). Accordingly, the mRNA levels of inflammatory cytokines IL-1β, IL-6 and TNF-α in anti-VCAM-1-treated animals were significantly lower than in IgG-treated animals after Ang II infusion (Figure 4D). In addition, we assessed the activation of proinflammatory pathways and found that the Ang II infusion-induced increases in the protein levels of VLA-4, p-IKKα and p-p65 were greatly attenuated in anti-VCAM-1-treated mice (Figure 4E). These findings indicate that VCAM-1 promotes cardiac inflammatory by increasing the infiltration of VLA-4^+^ macrophages into the heart.

### Blocking VCAM-1 reduces Ang II-induced DNA damage and oxidative stress

**FIGURE 5:**
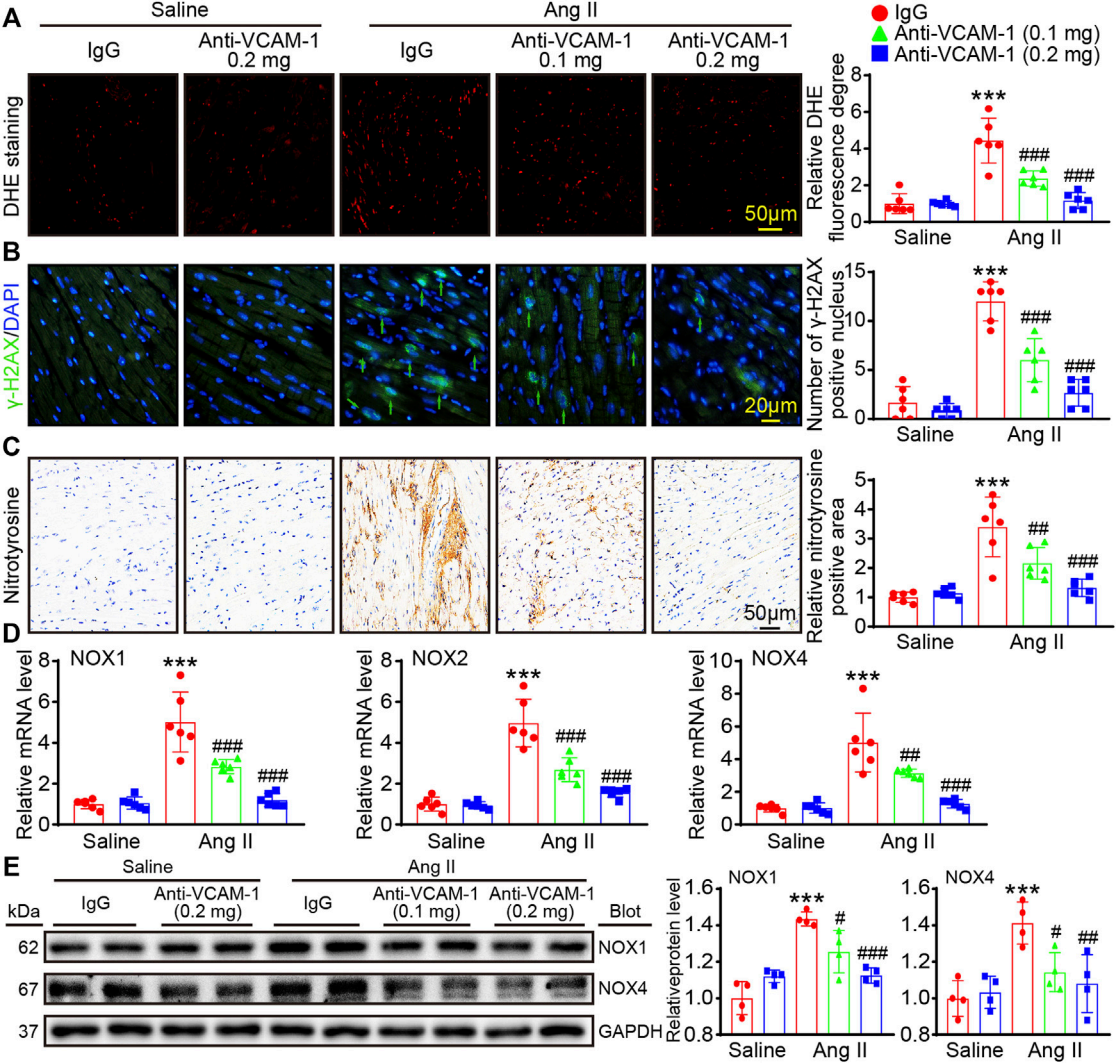
Blockade of VCAM-1 reduces Ang II-induced oxidative stress and DNA damage. **(A)** Immunofluorescence staining of heart sections (left). Scale bar: 50 μm. Analysis of the DHE fluorescence intensity (right, *n* = 6) **(B)** Immunofluorescence staining of heart sections with an anti-γ-H2AX antibody (left). Scale bar: 20 μm. Analysis of γ-H2AX-positive nuclei (right, *n* = 6) **(C)** Immunohistochemical staining of heart sections with an anti-nitrotyrosine antibody (left). Scale bar: 50 μm. Analysis of nitrotyrosine-positive areas (right, *n* = 6) **(D)** qPCR analysis of NOX1, NOX2 and NOX4 expression in the heart (*n* = 6) **(E)** Immunoblot analysis of NOX1 and NOX4 (left) and analysis the levels of these proteins (right, *n* = 4). The data are expressed as the M ± SD, and n represents the number of animals. ***p < 0.001 vs saline + IgG group. ^#^ *p* < 0.05, ^##^ *p* < 0.01 and ^###^ *p* < 0.001 vs Ang II + IgG group.

Oxidative stress plays a significant role in cardiac remodeling. Therefore, DHE staining was performed to measure ROS levels in heart and found that Ang II-induced augment in ROS level, which was evidenced by an increase in the DHE fluorescence intensity, was markedly dose-dependently suppressed in anti-VCAM-1-treated animals compared with IgG-treated animals (Figure 5A). Moreover, immunostaining for γ-H2AX revealed that Ang II increased γ-H2AX level in nuclei in IgG-treated animals, and this effect was dose-dependently abolished in anti-VCAM-1-treated animals (Figure 5B). Furthermore, we performed immunohistochemical staining to measure the level of nitrotyrosine (an important indicator of the oxidative stress level). As expected, the nitrotyrosine-positive area as was dose-dependently decreased in anti-VCAM-1-treated animals compared with IgG-treated animals after Ang II treatment (Figure 5C). Consistently, the Ang II-caused increase of NADPH oxidase isoforms (NOX1, NOX2 or NOX4), the major source of ROS, was also dose-dependently abrogated by anti-VCAM-1 treatment (Figures 5D,E). These results suggest that increased VCAM-1 expression aggravates oxidative, stress leading to DNA damage.

### Blocking VCAM-1 inhibits BMMs adhesion and migration, and alleviates cardiomyocyte hypertrophy and myofibroblast activation *in vitro*

**FIGURE 6:**
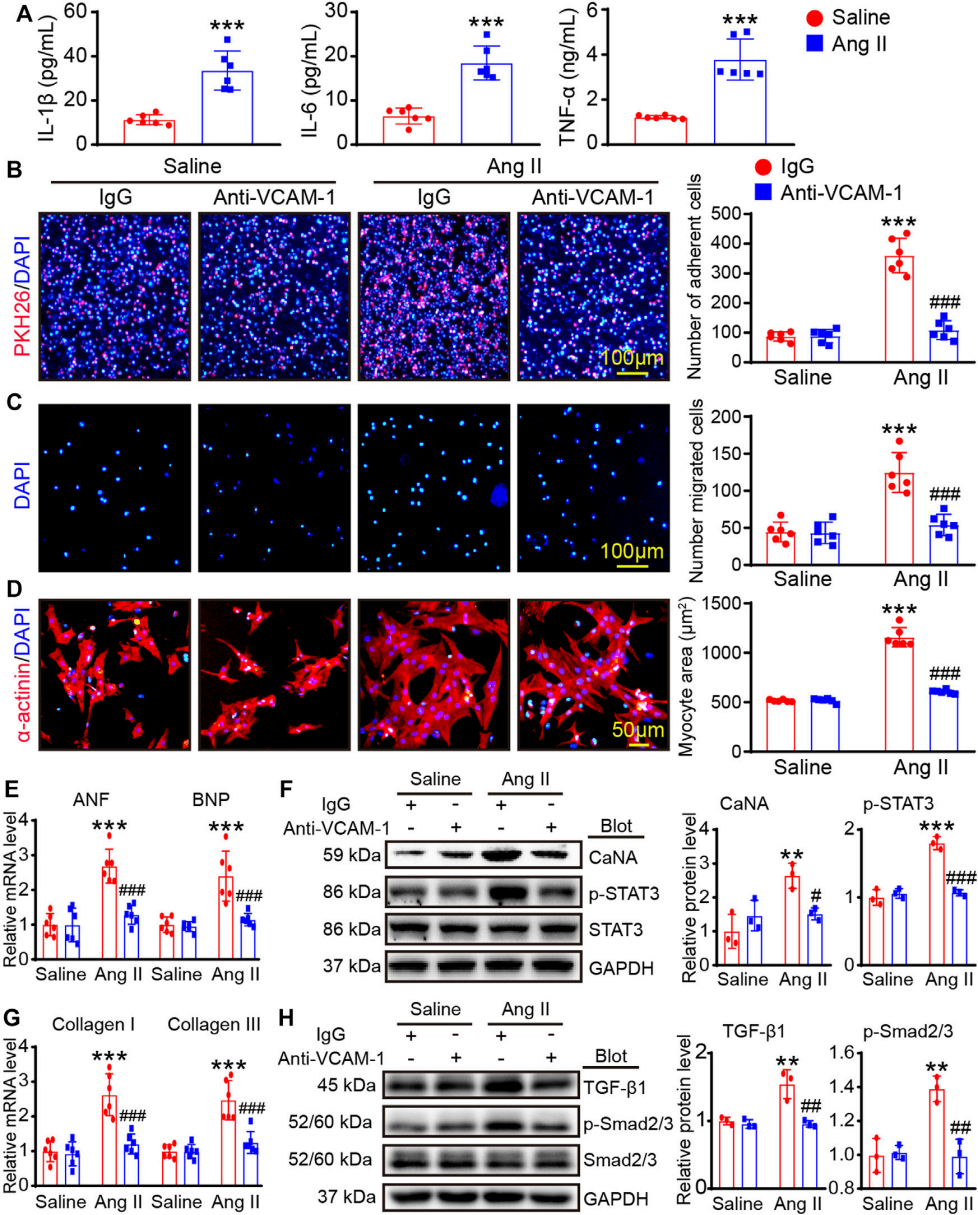
Blocking VCAM-1 inhibits macrophage adhesion and migration and reduces cardiomyocyte hypertrophy and myofibroblast activation *in vitro*. **(A)** The bone marrow-derived macrophages (BMMs) were stimulated with Ang II (100 nM) or saline for 24 h. ELISA analysis of the IL-1β, IL-6 and TNF-α levels (*n* = 6) **(B)** HUVECs were pretreated an anti-VCAM-1 antibody (5 μg/ml) or IgG control for 2 h and then stimulated with Ang II (100 nM) or saline for an additional 24 h. Adhesion of PKH26-labeled BMMs (red, left, scale bar: 100 μm) and analysis of adherent cells (right, *n* = 6 fields) **(C)** Migration of BMMs (left). Scale bar: 100 μm. Analysis of migrated cells (right, *n* = 6 fields) **(D)** HUVECs and BMMs were co-cultured and treated with anti-VCAM-1 (5 μg/ml) and Ang II (100 nM) for 24 h. The supernatants were collected and added to CMs or CFs for 24 h. Cardiomyocyte size was determined using immunostaining with anti-α-actinin antibody. **(E)** The mRNA levels of ANF and BNP in CMs were analyzed by qPCR analysis (*n* = 6). **(F)** The protein levels of CaNA and p-STAT3 in lysates from CMs were determined by immunoblotting analysis (left), and quantification of relative protein level (right, *n* = 3 independent experiments). **(G)** The mRNA levels of collagen I and collagen III in CFs were analyzed by qPCR analysis (*n* = 6). **(H)** The protein levels of TGF-β1 and p-Smad2/3 in lysates from CFs were evaluated by immunoblotting analysis (left), and quantification of relative protein level (right, *n* = 3 independent experiments).

VCAM-1 has significant effects in mediating leukocytes infiltration into heart tissue (Takamatsu, 2018). Firstly, we performed ELISA to evaluate the concentration of the IL-1β, IL-6 and TNF-α in cultured VLA4^+^ macrophages after saline or Ang II stimulation, and the results showed that Ang II markedly upregulated the levels of these cytokines (Figure 6A). Then, we performed a transwell assay to test whether blocking VCAM-1 can affect BMMs adhesion and migration to HUVECs. The results showed that Ang II markedly upregulated the number of PKH-26-labeled adhered macrophages and migrated macrophages in the IgG-treated group, but this increase was dose-dependently suppressed by anti-VCAM-1 treatment (Figures 6B,C). Furthermore, we tested the effect of BMMs on neonatal rat cardiomyocytes (CMs) or cardiac fibroblasts (CFs). Our results indicated that pretreatment of BMMs with the anti-VCAM-1 obviously inhibited Ang II-caused cardiomyocyte hypertrophy, as indicated by the decrease in CM size, the mRNA levels of ANF and BNP, and the protein levels of CaNA and p-STAT3 compared with IgG-treated group (Figures 6D–F). Similarly, pretreatment of BMMs with the anti-VCAM-1 also markedly reduced Ang II-stimulated differentiation of CFs, as showed by the reduction in the mRNA levels of collagen I and collagen III, as well as the protein levels of TGF-β1 and p-Smad2/3 compared with IgG-treated group (Figures 6G,H).

## Discussion

**FIGURE 7:**
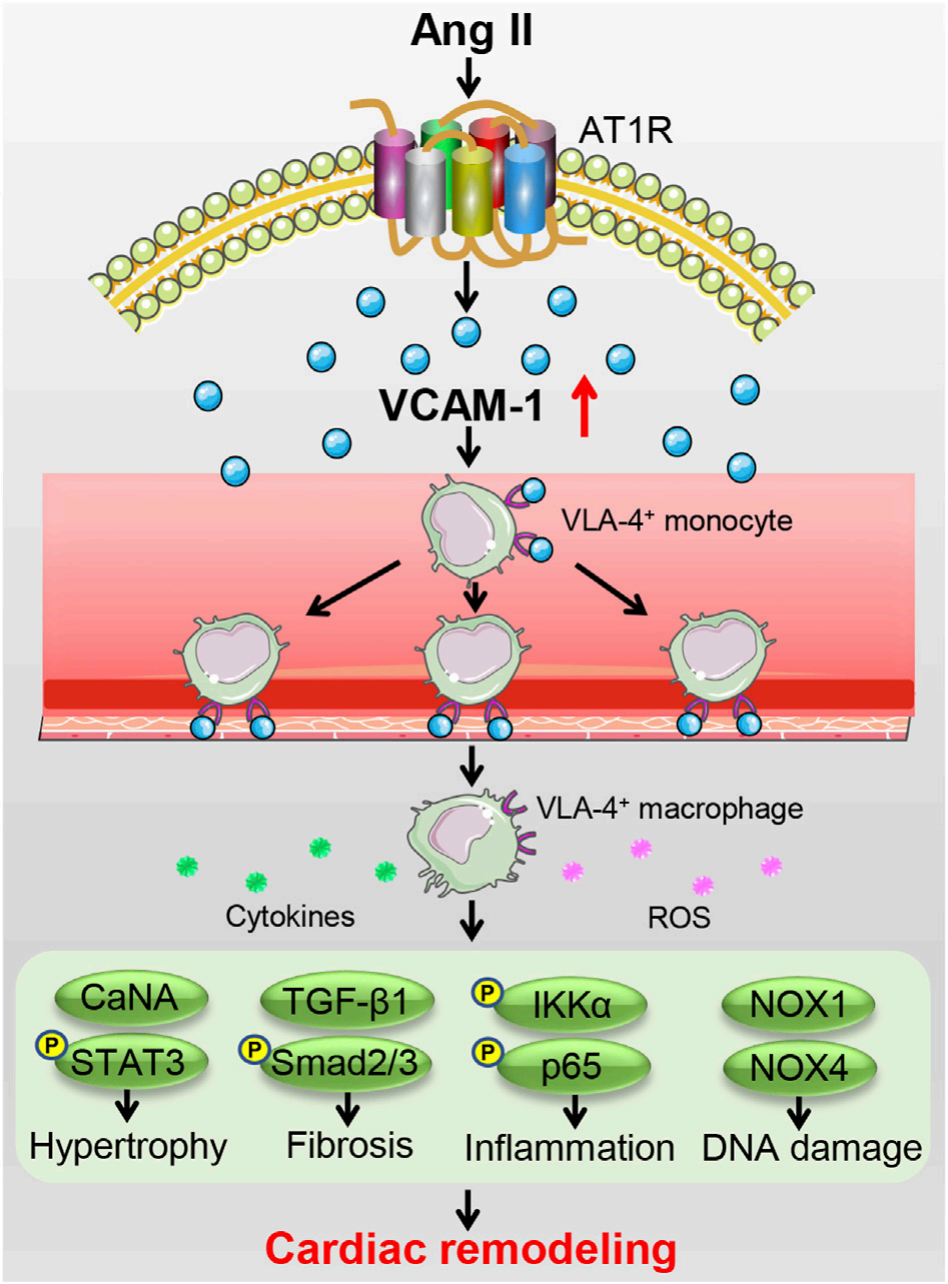
A working model of the mechanism by which VCAM-1 regulates Ang II-induced cardiac remodeling. Ang II infusion upregulates VCAM-1 expression, which promotes VLA-4^+^ macrophage adhesion to the endothelium and infiltration into the heart, inducing the release of abundant proinflammatory cytokines and ROS to activate multiple signaling pathways, thereby leading to cardiomyocyte hypertrophy, myofibroblast activation, and subsequent cardiac remodeling. Conversely, selective blockade of VCAM-1 effectively prevents these effects.

This study demonstrates that blocking VCAM-1 attenuates Ang II-induced cardiac remodeling in mice. Ang II infusion significantly upregulates cardiac VCAM-1 level that promotes adhesion of VLA-4^+^ monocytes to the endothelium and their subsequent transendothelial migration and differentiation into mature macrophages. These cells produce various proinflammatory cytokines and ROS that activates multiple signaling pathways (CaNA/STAT3, TGF-β1/Smad2/3, IKKɑ/NF-kB, and NOX1/4), thereby leading to cardiac hypertrophy, fibrosis, inflammation and DNA damage (cardiac remodeling). On the contrary, blocking VCAM-1 can effectively attenuate these effects. A working model is shown in the diagram (Figure 7).

Pathological cardiac remodeling is characterized by changes in cardiac structure and function in response to various injuries or risk factors, such as ischemia/reperfusion (I/R) injury, pressure overload, and neurohormonal activation (Aimo et al., 2019). Multiple mechanisms are involved in pathological remodeling, including abnormal interactions of cardiomyocytes and noncardiomyocytes, oxidative stress, activation of the endoplasmic reticulum, alterations in autophagy induction, and impairment of metabolism (Nakamura and Sadoshima, 2018). Interestingly, inflammation is an important contributor to pathological cardiac remodeling in a number of cardiac diseases. Both chemokines and cell adhesion molecules participate in CVDs by regulating macrophage mobilization, adhesion and migration. Our previous data indicate that CXCL1-CXCR2 signaling acts pivotal parts in preventing Ang II- or DOCA-salt-caused infiltration of macrophages and subsequent cardiovascular remodeling and dysfunction (Wang et al., 2016; Wang et al., 2018; Zhang et al., 2020). VCAM-1 ubiquitously expresses in diverse cardiovascular cell types, and its expression is induced under pathological conditions (Theiss et al., 2007; Troncoso et al., 2021). For example, VCAM-1 level is augmented in arteries in Ang II-induced hypertension and sheep mitral valves after myocardial infarction (Bartko et al., 2017; Yin et al., 2022). Moreover, the mRNA expression of VCAM-1 is significantly increased in patients with ischemic cardiomyopathy (Theiss et al., 2007). Our current data indicate that VCAM-1 levels are increased in the hearts of Ang II-infused mice and serum of patients with HF (Figure 1), which suggest that increased VCAM-1 may be involved in the pathogenesis of hypertensive cardiac remodeling.

VCAM-1 is the critical adhesion protein involved in the inflammatory response and various immunological diseases, and serves as a potential therapeutic target for these disorders. VCAM-1 expression is regulated by multiple factors, including the redundant production of ROS, overexpression of cytokines, cholesterol, oxidized lipoprotein, high glucose, and turbulent shear stress (Cook-Mills et al., 2011). Research data indicated that the hydrogen peroxide (400 mM) could regulate VCAM-1 expression through activating the transcription factor of NF-κB in aortic endothelial cells (Cook-Mills et al., 2011). In addition, the cytokine of TNF-α can also induce VCAM-1 expression in endothelial cells, which was suppressed by high levels of superoxide dismutase (Cook-Mills et al., 2011). Moreover, the VCAM-1-induced IL-1β activation will be blocked by antioxidants, including pyrrolidine dithiocarbamate, N-acetylcysteine, and α-tocopherol (Cook-Mills et al., 2011). It is reported that the interaction of disintegrin and ADAM1 could also promote VCAM-1 overexpress on the endothelial surface (Cook-Mills et al., 2011).

Several data have reported that the interactions between VCAM-1 and VLA-4 (α4β1) is crucial in the progression of autoimmune diseases (Kong et al., 2018). Moreover, activation of VCAM-1-VLA-4 signaling is associated with several types of CVDs, such as hypertensive and ischemic diseases, atherosclerosis, stroke, heart failure (HF), arrhythmias and AF (Gurtner et al., 1995; Kwee et al., 1995; Troncoso et al., 2021). For example, transplanted cardiac progenitor cells that express VCAM-1 can activate the AKT, ERK and p38 MAPK signaling pathways and consequently prevent oxidative stress-induced cardiomyocyte death, eventually attenuating cardiac remodeling and dysfunction after myocardial infarction (Matsuura et al., 2009; Troncoso et al., 2021). Moreover, VCAM-1 triggers monocyte migration and infiltration into the endothelium through VLA-4 in early atherosclerosis (Huo et al., 2001). In addition, increased VCAM-1 levels are important for the initiation of inflammatory and prothrombotic effects, which are essential for atrial thrombus development and the occurrence of postoperative AF (Verdejo et al., 2011; Troncoso et al., 2021). Recently, our results demonstrated that Ang II-caused VCAM-1 overexpression can promote the infiltration of macrophages in arteries, leading to endothelial dysfunction and hypertension (Yin et al., 2022). Here, our results further confirm that upregulation of VCAM-1 can promote the infiltration of VLA-4^+^ macrophages, which release abundant proinflammatory cytokines and ROS that activate multiple signaling pathways (CaNA/STAT3, TGF-β1/Smad2/3, IKKɑ/NF-kB, and NOX1/4), thereby resulting in cardiac remodeling. Inversely, blocking VCAM-1 suppresses these performances. Therefore, targeting VCAM-1 may be a new therapeutic strategy for hypertensive cardiac remodeling and HF. However, some limitations exist in current study. The preventive effect of VCAM-1 blockage against cardiac remodeling and dysfunction must be explored in female mice and VCAM-1-deficient mice. The precise mechanism by which Ang II increases VCAM-1 expression and whether VCAM-1 affects cardiac ion channels during Ang II infusion remain to be determined.

In conclusion, these data suggest that VCAM-1 participates in Ang II-induced cardiac structural remodeling. Our *in vivo* and *in vitro* results indicated that blocking VCAM-1 can effectively attenuate Ang II-induced hypertension and cardiac remodeling possibly by reducing VLA-4^+^ macrophage adhesion/migration and generation of IL-1β, IL6 and TNF-ɑ and ROS, as well as activation of multiple signaling pathway. Therefore, targeting VCAM-1 may represent a novel therapeutic approach for the treatment of hypertensive cardiac remodeling.

## Data Availability

The original contributions presented in the study are included in the article/supplementary material, further inquiries can be directed to the corresponding author.
