# Supplementary material for: Blocking VCAM-1 ameliorates hypertensive cardiac remodeling by impeding macrophage infiltration
Source: Front Pharmacol. 2022 Nov 17;13:1058268. doi: 10.3389/fphar.2022.1058268 (PMC9713306; doi:10.3389/fphar.2022.1058268)

**Raw data presented in our tables and figures**

**Figure 1A** Analysis of serum VCAM-1 levels in HF patients and control subjects by ELISA

| **Control** | | **HF** | |
| --- | --- | --- | --- |
| **Number** | **C(ng/ml)** | **Number** | **C(ng/ml)** |
| C1091-2 | 77.07 | HA32 | 768.57 |
| C1063-2 | 77.20 | HA26 | 762.29 |
| C1059-2 | 82.38 | HA46 | 755.74 |
| C1109-2 | 83.05 | S272-2 | 742.65 |
| C1141-2 | 91.78 | S87-2 | 693.17 |
| C1075-2 | 98.82 | HA39 | 692.01 |
| C1026-2 | 103.85 | S258-2 | 676.26 |
| C1094-2 | 106.96 | HF10 | 675.18 |
| C1197-2 | 130.07 | S312-2 | 670.54 |
| C1079-2 | 131.55 | S44-2 | 670.02 |
| C1052-2 | 135.19 | HF8 | 669.21 |
| C1140-2 | 135.42 | HA31 | 668.55 |
| C1071-2 | 135.94 | S100-2 | 667.72 |
| C1044-2 | 144.82 | S228-2 | 667.56 |
| C1088-2 | 147.71 | HF2 | 660.34 |
| C1168-2 | 151.99 | HA45 | 648.11 |
| C1136-2 | 153.37 | S26-2 | 643.04 |
| C1020-2 | 156.61 | S306-2 | 632.25 |
| C1200-2 | 159.94 | S199-2 | 626.36 |
| C1108-2 | 161.41 | S30-1 | 625.56 |
| C1025-2 | 164.71 | S81-2 | 621.99 |
| C1170-2 | 169.68 | HA23 | 606.72 |
| C1142-2 | 175.24 | S6-2 | 599.33 |
| C1121-2 | 179.34 | HA44 | 591.73 |
| C1120-2 | 186.22 | S334-2 | 589.56 |
| C1054-2 | 186.22 | S3-3 | 575.08 |
| C1047-2 | 186.51 | HF3 | 568.43 |
| C1116-2 | 186.86 | S305-2 | 562.63 |
| C1092-2 | 187.34 | HF5 | 539.42 |
| C1036-2 | 188.39 | S178-2 | 527.87 |

**Figure 1B** Analysis of serum BNP levels in HF patients and control subjects by ELISA

| **Control** | | **HF** | |
| --- | --- | --- | --- |
| **Number** | **C(pg/ml)** | **Number** | **C(pg/ml)** |
| C1091-2 | 27.83 | HA32 | 182.91 |
| C1063-2 | 22.08 | HA26 | 154.44 |
| C1059-2 | 31.91 | HA46 | 183.03 |
| C1109-2 | 29.25 | S272-2 | 145.85 |
| C1141-2 | 23.08 | S87-2 | 170.46 |
| C1075-2 | 34.94 | HA39 | 170.22 |
| C1026-2 | 35.82 | S258-2 | 199.78 |
| C1094-2 | 31.07 | HF10 | 149.52 |
| C1197-2 | 28.34 | S312-2 | 105.88 |
| C1079-2 | 30.26 | S44-2 | 210.43 |
| C1052-2 | 27.77 | HF8 | 199.55 |
| C1140-2 | 15.67 | HA31 | 160.69 |
| C1071-2 | 27.56 | S100-2 | 166.19 |
| C1044-2 | 28.55 | S228-2 | 146.15 |
| C1088-2 | 20.94 | HF2 | 108.43 |
| C1168-2 | 21.13 | HA45 | 170.44 |
| C1136-2 | 22.40 | S26-2 | 207.82 |
| C1020-2 | 15.48 | S306-2 | 205.74 |
| C1200-2 | 34.69 | S199-2 | 195.37 |
| C1108-2 | 14.30 | S30-1 | 195.29 |
| C1025-2 | 12.66 | S81-2 | 207.78 |
| C1170-2 | 20.96 | HA23 | 224.79 |
| C1142-2 | 28.16 | S6-2 | 184.28 |
| C1121-2 | 22.41 | HA44 | 229.89 |
| C1120-2 | 17.48 | S334-2 | 193.73 |
| C1054-2 | 20.98 | S3-3 | 202.19 |
| C1047-2 | 22.45 | HF3 | 216.89 |
| C1116-2 | 21.30 | S305-2 | 145.58 |
| C1092-2 | 27.43 | HF5 | 192.24 |
| C1036-2 | 16.75 | S178-2 | 223.01 |

**Figure 1C** qPCR analysis of VCAM-1 mRNA levels in heart tissues

|  | **VCAM-1** | **Normalization** |
| --- | --- | --- |
| Saline | 25.76 | 0.80 |
| Saline | 23.33 | 1.31 |
| Saline | 23.79 | 1.09 |
| Saline | 23.12 | 1.23 |
| Saline | 23.12 | 0.74 |
| Saline | 23.31 | 0.83 |
| Ang II | 22.85 | 1.89 |
| Ang II | 23.07 | 2.67 |
| Ang II | 22.92 | 0.95 |
| Ang II | 23.81 | 2.63 |
| Ang II | 22.55 | 1.41 |
| Ang II | 23.42 | 1.74 |

**Figure 1D** Immunoblot analysis of VCAM-1 protein levels in heart tissues


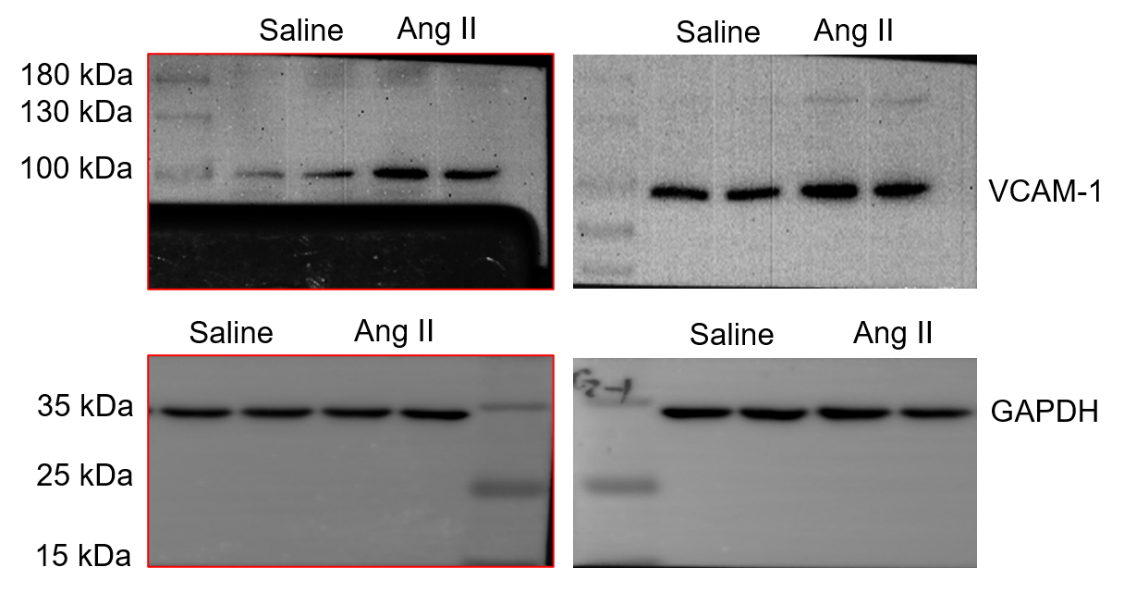


|  | Saline | Divided by gapdh | Normalization | Ang II | Divided by gapdh | Normalization |
| --- | --- | --- | --- | --- | --- | --- |
| VCAM-1 | 10740.27 | 0.27 | 0.52 | 37483.48 | 0.93 | 1.75 |
|  | 18156.07 | 0.46 | 0.87 | 29281.60 | 0.73 | 1.37 |
|  | 27715.24 | 0.70 | 1.33 | 38959.97 | 0.97 | 1.82 |

**Figure 1E:** Immunohistochemical staining of VCAM-1


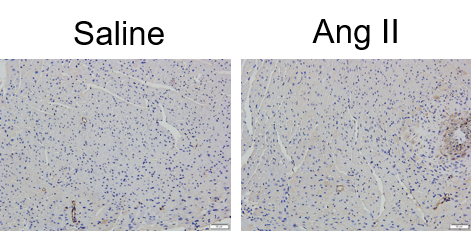


**Figure 2B:** Blood pressure

|  | Saline + IgG | Saline + Anti-VCAM1 (0.2mg) | Ang II + IgG | Ang II + Anti-VCAM1 (0.1mg) | Ang II + Anti-VCAM1 (0.2mg) |
| --- | --- | --- | --- | --- | --- |
| C1 | 93 | 93 | 92 | 93 | 94 |
| A1 | 95 | 94 | 118 | 107 | 96 |
| A3 | 93 | 93 | 137 | 113 | 95 |
| A5 | 93 | 97 | 143 | 118 | 105 |
| A7 | 94 | 92 | 146 | 121 | 107 |
| A9 | 94 | 91 | 147 | 125 | 105 |
| A11 | 92 | 94 | 156 | 126 | 106 |
| A13 | 93 | 94 | 169 | 133 | 102 |

**Figure 2C:** Echocardiography


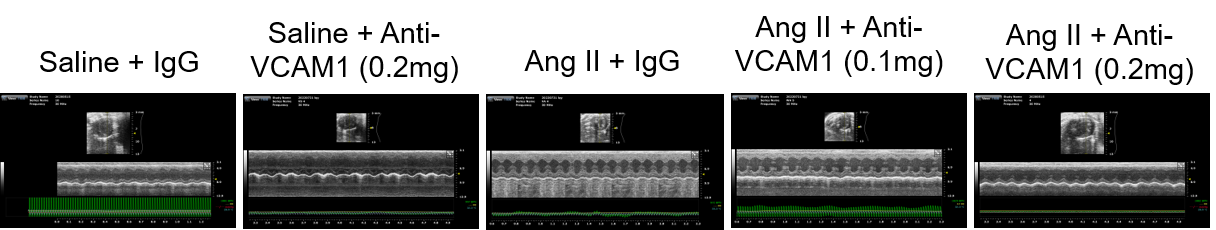


**Figure 2D** H&E staining


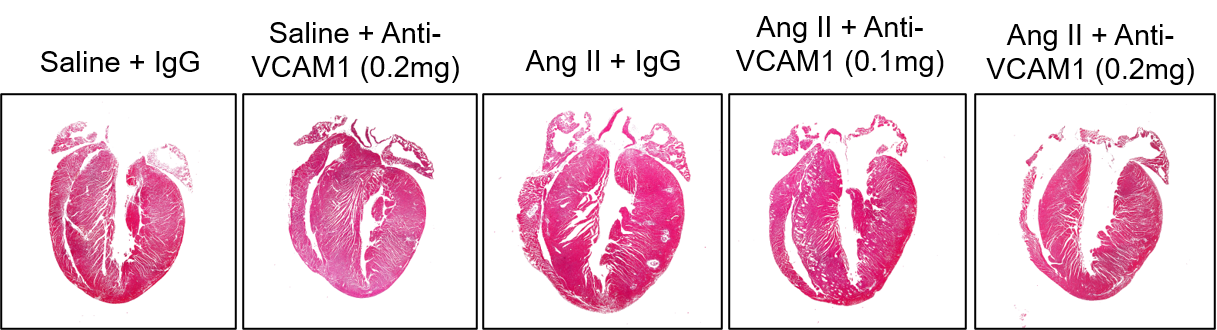


**Figure 2E** WGA staining

**
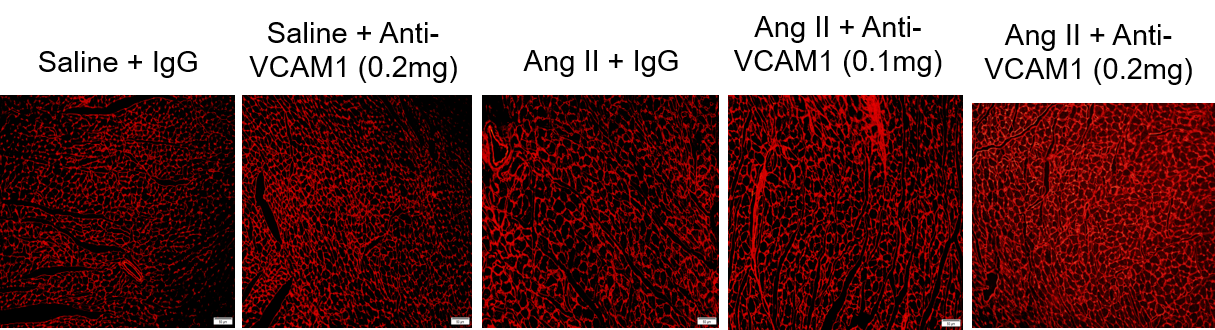
**

**Figure 2F** qPCR analysis of ANF, BNP and MYH7 levels in the heart

|  | ANF | BNP | MYH7 |
| --- | --- | --- | --- |
| Saline + IgG | 19.68 | 22.92 | 30.74 |
|  | 19.12 | 22.21 | 30.60 |
|  | 19.75 | 22.00 | 30.20 |
|  | 19.19 | 22.42 | 30.28 |
|  | 20.83 | 24.69 | 31.40 |
|  | 20.47 | 24.86 | 30.86 |
| Saline+Anti-VCAM1 (0.2mg) | 18.81 | 21.96 | 29.56 |
|  | 18.88 | 21.48 | 29.31 |
|  | 18.45 | 20.99 | 28.50 |
|  | 18.22 | 21.95 | 29.38 |
|  | 17.60 | 21.45 | 29.04 |
|  | 18.07 | 21.74 | 28.50 |
| Ang II + IgG | 17.75 | 20.81 | 28.51 |
|  | 17.54 | 21.95 | 28.71 |
|  | 18.97 | 21.97 | 28.92 |
|  | 18.98 | 21.86 | 29.90 |
|  | 18.54 | 21.68 | 27.87 |
|  | 19.16 | 22.18 | 28.91 |
| Ang II + Anti-VCAM1 (0.1mg) | 18.47 | 21.45 | 28.14 |
|  | 18.05 | 21.86 | 28.05 |
|  | 18.59 | 21.47 | 28.37 |
|  | 17.49 | 21.18 | 27.94 |
|  | 17.79 | 20.67 | 28.26 |
|  | 18.28 | 21.32 | 28.64 |
| Ang II + Anti-VCAM1 (0.2mg) | 19.81 | 23.26 | 30.54 |
|  | 19.82 | 22.52 | 30.76 |
|  | 19.67 | 23.02 | 30.60 |
|  | 20.01 | 22.35 | 29.84 |
|  | 19.97 | 23.20 | 30.46 |
|  | 19.16 | 22.72 | 30.13 |

**Figure 2G** Immunoblot analysis of cardiac calcineurin A (CaNA) and p-STAT3


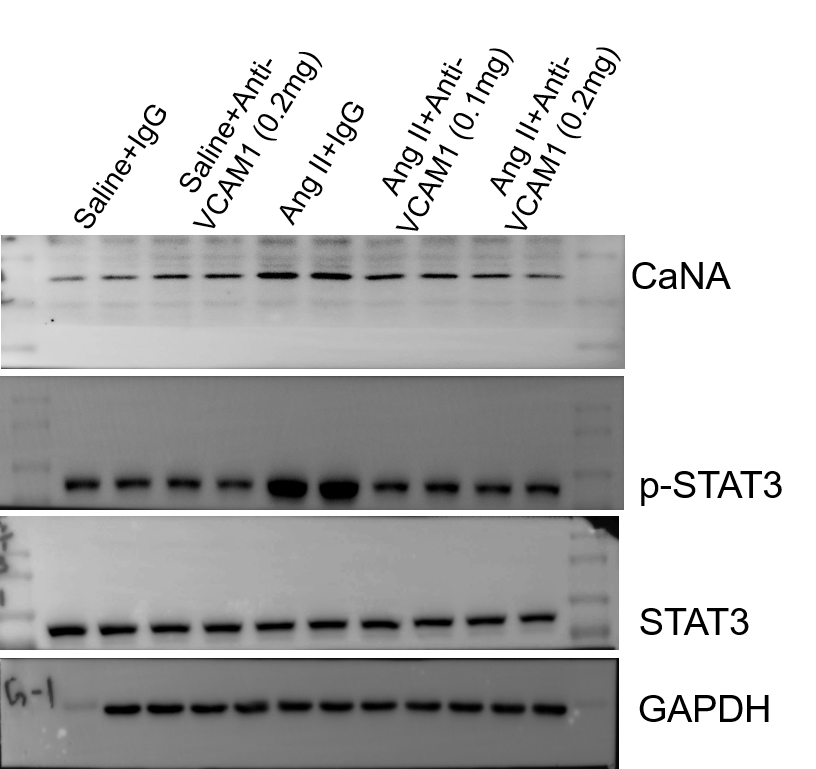


**Figure 3A** Masson’s trichrome staining


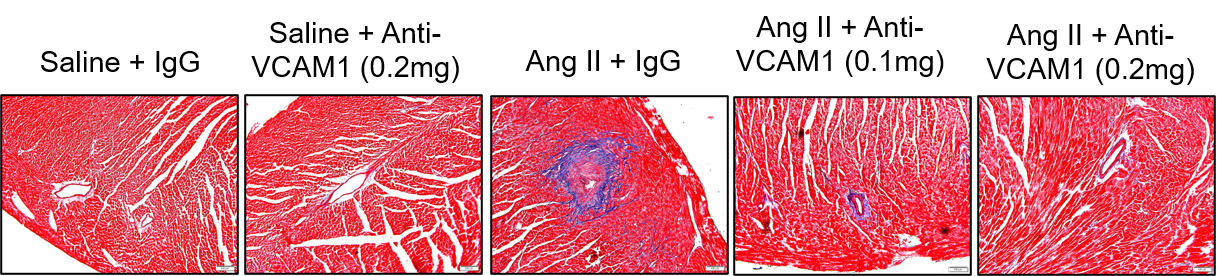


**Figure 3B** Immunofluorescence staining of heart sections with an anti-collagen III


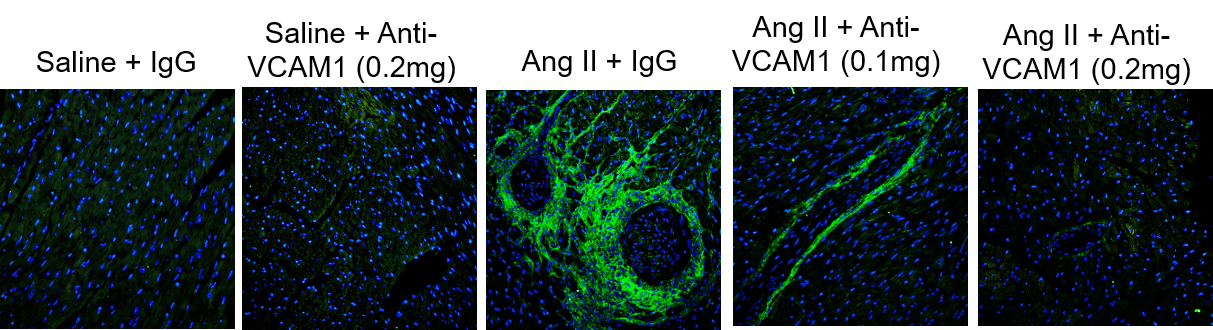


**Figure 3C** Immunohistochemical staining of heart sections with an anti-α-SMA antibody


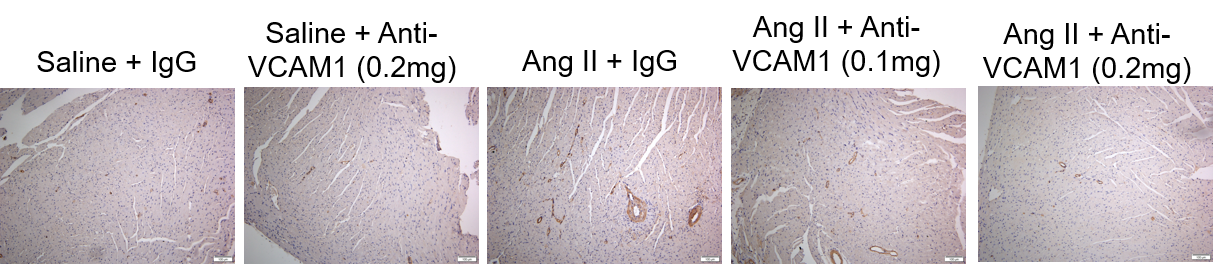


**Figure 3D** qPCR analysis of collagen I, collagen III and α-SMA expression

|  | collagen I | collagen III | α-SMA |
| --- | --- | --- | --- |
| Saline + IgG | 25.12 | 23.60 | 24.45 |
|  | 24.31 | 23.43 | 23.96 |
|  | 24.70 | 23.96 | 23.46 |
|  | 25.29 | 23.30 | 23.62 |
|  | 26.02 | 24.60 | 24.86 |
|  | 26.85 | 24.65 | 24.84 |
| Saline+Anti-VCAM1 (0.2mg) | 24.05 | 22.52 | 22.41 |
|  | 24.15 | 22.67 | 23.07 |
|  | 23.14 | 22.22 | 21.95 |
|  | 24.27 | 22.25 | 22.65 |
|  | 24.31 | 21.97 | 22.46 |
|  | 23.93 | 22.62 | 22.97 |
| Ang II + IgG | 17.75 | 20.81 | 28.51 |
|  | 17.54 | 21.95 | 28.71 |
|  | 18.97 | 21.97 | 28.92 |
|  | 18.98 | 21.86 | 29.90 |
|  | 18.54 | 21.68 | 27.87 |
|  | 19.16 | 22.18 | 28.91 |
| Ang II + Anti-VCAM1 (0.1mg) | 23.22 | 21.79 | 22.15 |
|  | 23.06 | 21.68 | 22.19 |
|  | 24.01 | 22.40 | 22.65 |
|  | 24.59 | 22.98 | 23.27 |
|  | 23.56 | 22.10 | 22.05 |
|  | 24.38 | 23.11 | 28.64 |
| Ang II + Anti-VCAM1 (0.2mg) | 23.67 | 22.92 | 22.95 |
|  | 23.67 | 21.89 | 22.03 |
|  | 23.73 | 22.42 | 22.72 |
|  | 23.24 | 22.39 | 22.17 |
|  | 23.46 | 21.40 | 21.77 |
|  | 23.71 | 22.03 | 21.99 |

**Figure 3E** Immunoblot analysis of TGF-β1 and p-Smad2/3


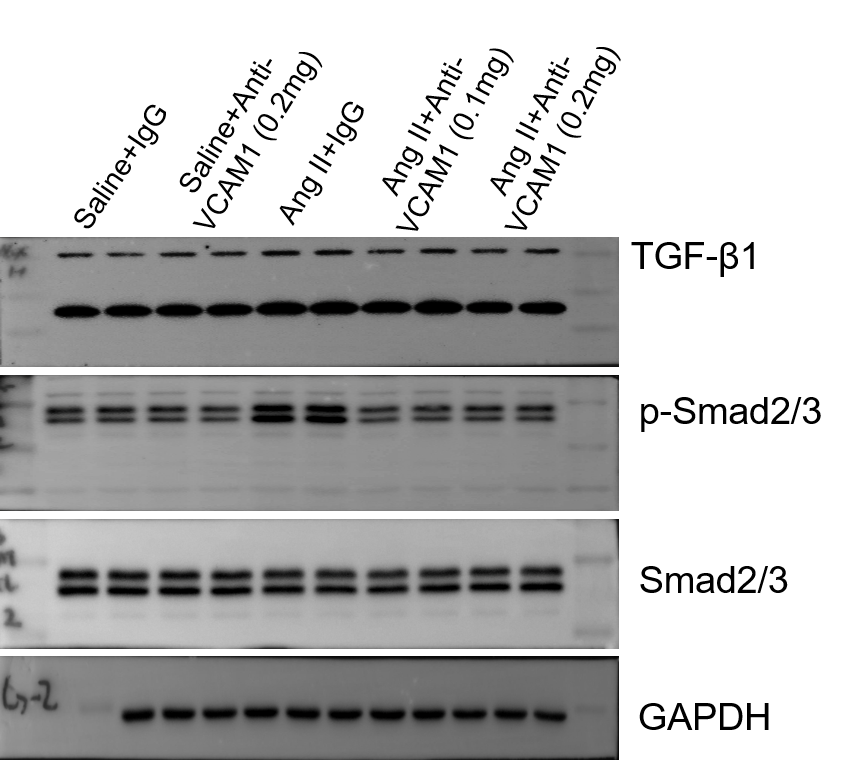


**Figure 4A** H&E staining


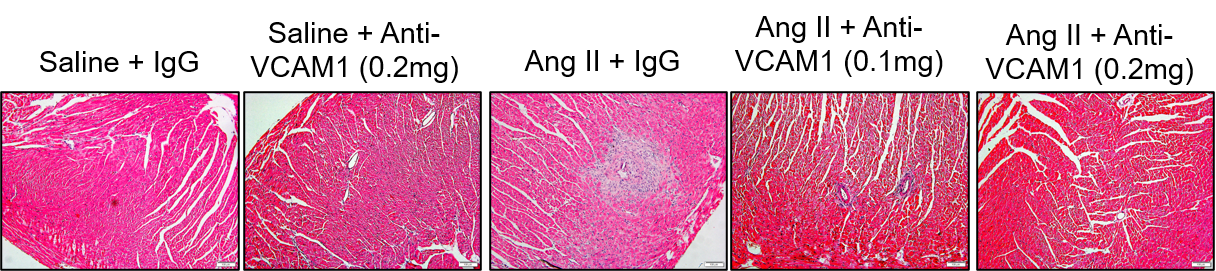


**Figure 4B** Immunohistochemical staining of heart sections with anti-CD68 antibody


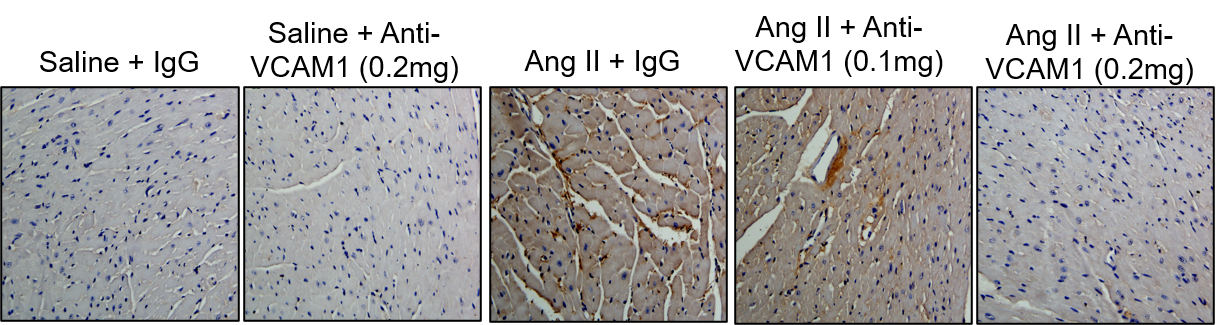


**Figure 4C** Immunofluorescence staining of heart sections with an anti-VLA-4 antibody


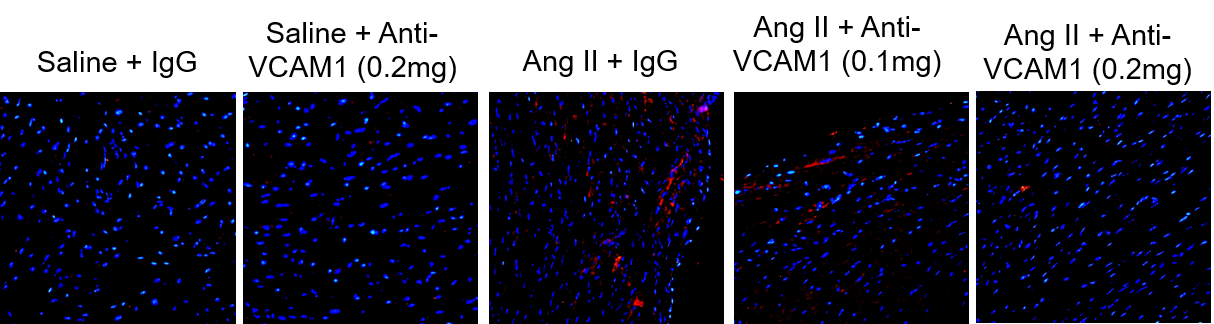


**Figure 4D** qPCR analysis of IL-1β, IL-6 and TNF-α

|  | IL-1β | IL-6 | TNF-α |
| --- | --- | --- | --- |
| Saline + IgG | 31.18 | 32.39 | 33.36 |
|  | 31.93 | 32.06 | 33.17 |
|  | 31.61 | 32.07 | 33.00 |
|  | 32.03 | 31.75 | 33.13 |
|  | 32.71 | 32.84 | 34.80 |
|  | 32.39 | 32.72 | 34.04 |
| Saline+Anti-VCAM1 (0.2mg) | 31.20 | 31.23 | 32.76 |
|  | 30.37 | 30.85 | 32.92 |
|  | 31.42 | 29.73 | 31.30 |
|  | 30.16 | 31.06 | 32.17 |
|  | 29.65 | 31.11 | 31.48 |
|  | 30.93 | 30.90 | 31.82 |
| Ang II + IgG | 29.81 | 29.93 | 31.25 |
|  | 29.76 | 30.27 | 31.65 |
|  | 31.38 | 30.97 | 32.93 |
|  | 30.53 | 30.26 | 31.87 |
|  | 30.07 | 30.94 | 32.17 |
|  | 30.79 | 30.41 | 32.78 |
| Ang II + Anti-VCAM1 (0.1mg) | 29.72 | 30.85 | 32.38 |
|  | 30.81 | 30.18 | 31.46 |
|  | 29.69 | 30.37 | 31.79 |
|  | 29.60 | 29.40 | 31.38 |
|  | 29.69 | 29.62 | 31.57 |
|  | 29.65 | 29.84 | 31.55 |
| Ang II + Anti-VCAM1 (0.2mg) | 31.38 | 31.92 | 33.84 |
|  | 30.77 | 32.14 | 33.36 |
|  | 31.59 | 32.23 | 33.24 |
|  | 31.98 | 31.93 | 33.62 |
|  | 31.40 | 31.78 | 33.27 |
|  | 31.39 | 31.89 | 33.95 |

**Figure 4E** Immunoblot analysis of VLA-4, p-IKKα and p-p65


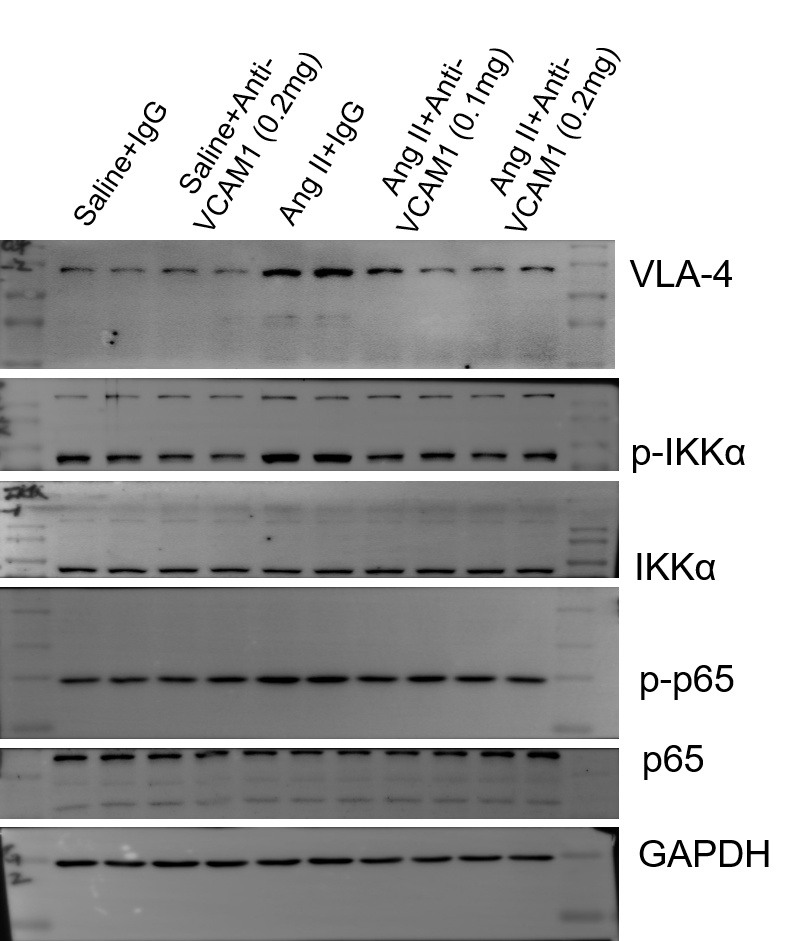


**Figure 5A** DHE staining


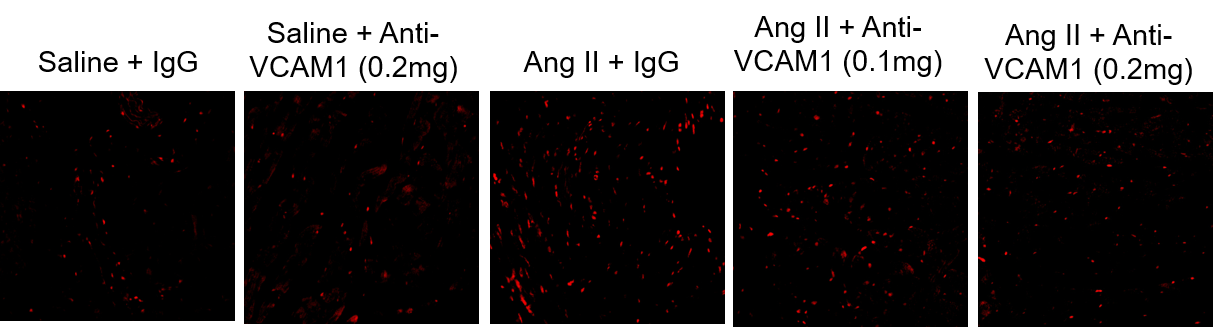


**Figure 5B** Immunofluorescence staining of heart sections with an anti-γ-H2AX antibody


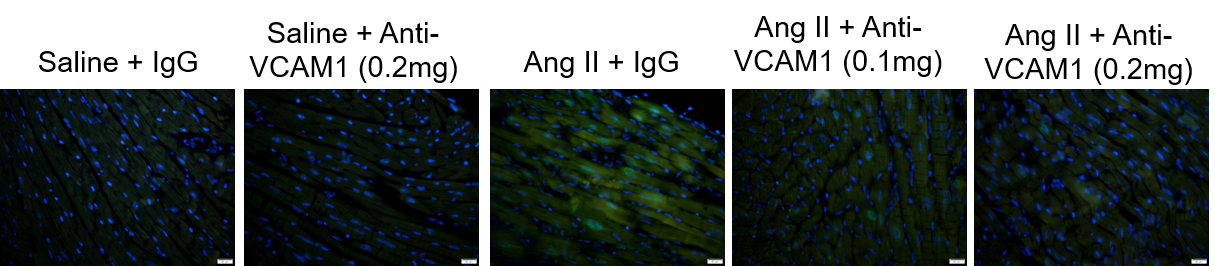


**Figure 5C** Immunohistochemical staining of heart sections with an anti-nitrotyrosine antibody


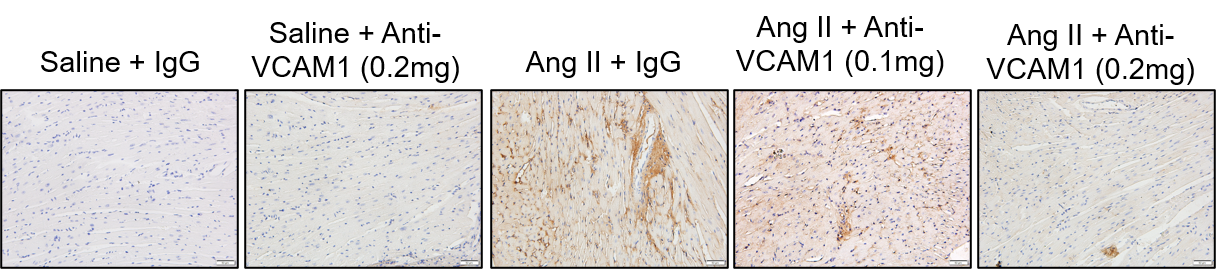


**Figure 5D** qPCR analysis of NOX1, NOX2 and NOX4

|  | NOX1 | NOX2 | NOX4 |
| --- | --- | --- | --- |
| Saline + IgG | 36.45 | 33.48 | 31.89 |
|  | 36.87 | 33.70 | 32.19 |
|  | 36.45 | 33.08 | 31.53 |
|  | 36.69 | 33.08 | 31.68 |
|  | 37.94 | 33.88 | 33.15 |
|  | 37.35 | 33.75 | 32.53 |
| Saline+Anti-VCAM1 (0.2mg) | 35.36 | 32.44 | 30.07 |
|  | 35.20 | 32.49 | 31.12 |
|  | 34.75 | 31.15 | 30.81 |
|  | 35.79 | 32.37 | 30.81 |
|  | 35.75 | 32.04 | 30.94 |
|  | 35.77 | 31.92 | 30.52 |
| Ang II + IgG | 34.99 | 30.95 | 29.85 |
|  | 34.40 | 30.91 | 30.03 |
|  | 35.64 | 32.04 | 29.90 |
|  | 34.76 | 32.23 | 31.20 |
|  | 35.04 | 30.94 | 30.08 |
|  | 35.65 | 32.32 | 30.73 |
| Ang II + Anti-VCAM1 (0.1mg) | 34.73 | 31.20 | 30.05 |
|  | 34.67 | 31.67 | 29.66 |
|  | 34.93 | 31.60 | 29.78 |
|  | 34.36 | 30.55 | 29.59 |
|  | 34.20 | 31.03 | 29.36 |
|  | 35.02 | 31.38 | 29.76 |
| Ang II + Anti-VCAM1 (0.2mg) | 36.84 | 33.41 | 31.97 |
|  | 36.39 | 32.76 | 31.66 |
|  | 36.26 | 32.74 | 31.53 |
|  | 36.92 | 32.48 | 31.50 |
|  | 36.91 | 32.59 | 31.95 |
|  | 37.08 | 32.91 | 32.39 |

**Figure 5E** Immunoblot analysis of NOX1 and NOX4


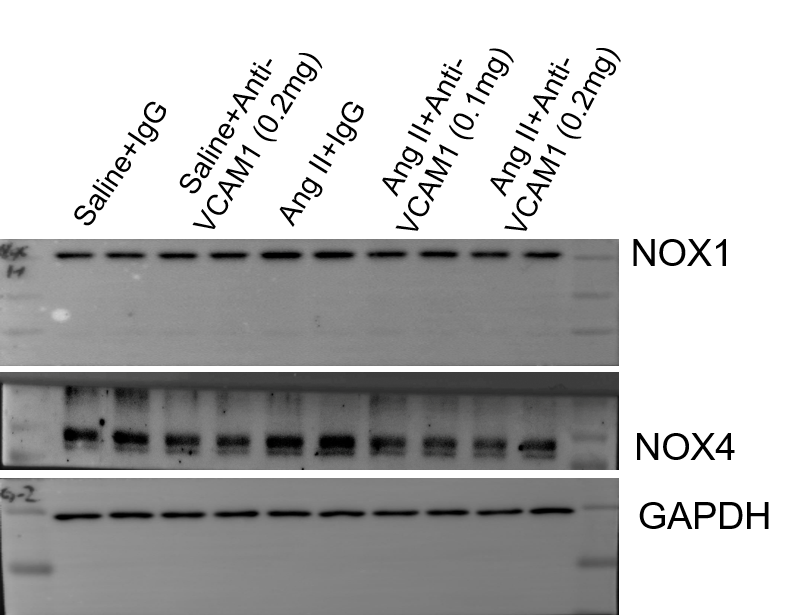


**Figure 6A** ELISA analysis of IL-1β, IL-6 and TNF-α

|  | IL-1β | IL-6 | TNF-α |
| --- | --- | --- | --- |
| Saline | 10.194 | 3.35 | 1.329 |
|  | 8.808 | 7.52 | 1.157 |
|  | 10.813 | 6.05 | 1.303 |
|  | 9.895 | 7.66 | 1.183 |
|  | 14.942 | 8.43 | 1.200 |
|  | 13.086 | 5.93 | 1.144 |
|  | 10.194 | 3.35 | 1.329 |
| Ang II | 47.649 | 15.21 | 3.171 |
|  | 25.283 | 21.25 | 3.209 |
|  | 24.995 | 17.15 | 3.254 |
|  | 35.777 | 15.82 | 3.140 |
|  | 28.910 | 16.56 | 5.020 |
|  | 38.681 | 24.96 | 4.901 |

**Figure 6B** Adhesion of PKH26-labeled bone marrow monocytes


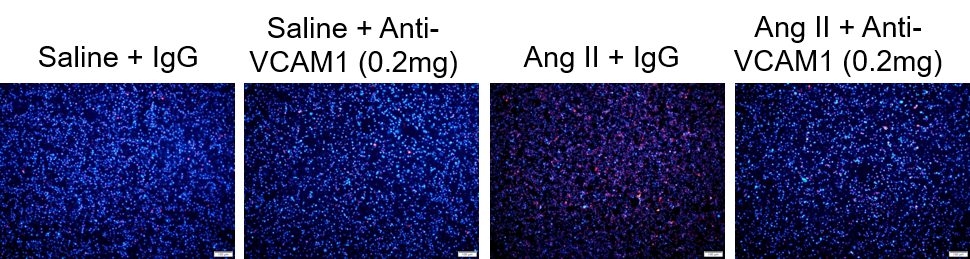


**Figure 6C** Migration of BMMs


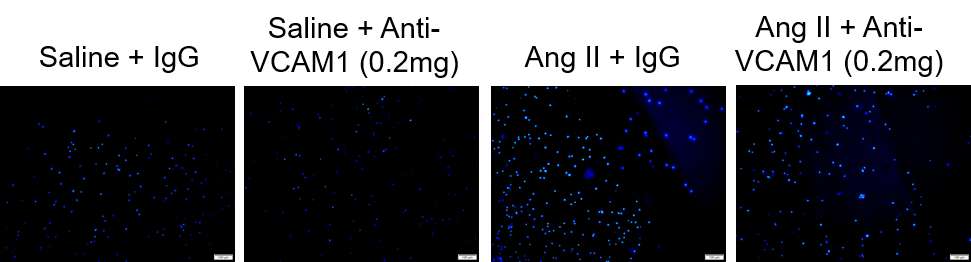


**Figure 6D** α-actinin immunostaining of cardiomyocyte


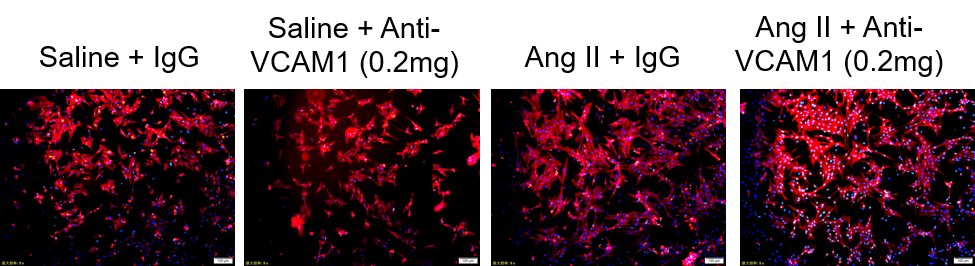


**Figure 6E** The mRNA levels of ANF and BNP in CMs

|  | ANF | BNP |
| --- | --- | --- |
| Saline+IgG | 0.91 | 1.26 |
|  | 1.47 | 1.05 |
|  | 0.70 | 0.74 |
|  | 1.23 | 0.80 |
|  | 1.05 | 0.91 |
|  | 0.64 | 1.25 |
|  | 0.91 | 1.26 |
| Saline+Anti-VCAM-1 | 1.46 | 0.72 |
|  | 1.25 | 0.98 |
|  | 1.56 | 0.91 |
|  | 0.45 | 1.03 |
|  | 0.60 | 1.14 |
|  | 0.63 | 0.92 |
| Ang II+IgG | 2.47 | 1.90 |
|  | 2.28 | 3.54 |
|  | 3.57 | 1.61 |
|  | 2.78 | 2.63 |
|  | 2.26 | 1.94 |
|  | 2.78 | 2.80 |
| Ang II+Anti-VCAM-1 | 1.27 | 1.14 |
|  | 1.62 | 0.94 |
|  | 1.30 | 1.11 |
|  | 1.53 | 1.18 |
|  | 1.12 | 1.47 |
|  | 0.87 | 1.03 |

**Figure 6F** Immunoblot of CaNA and p-STAT3


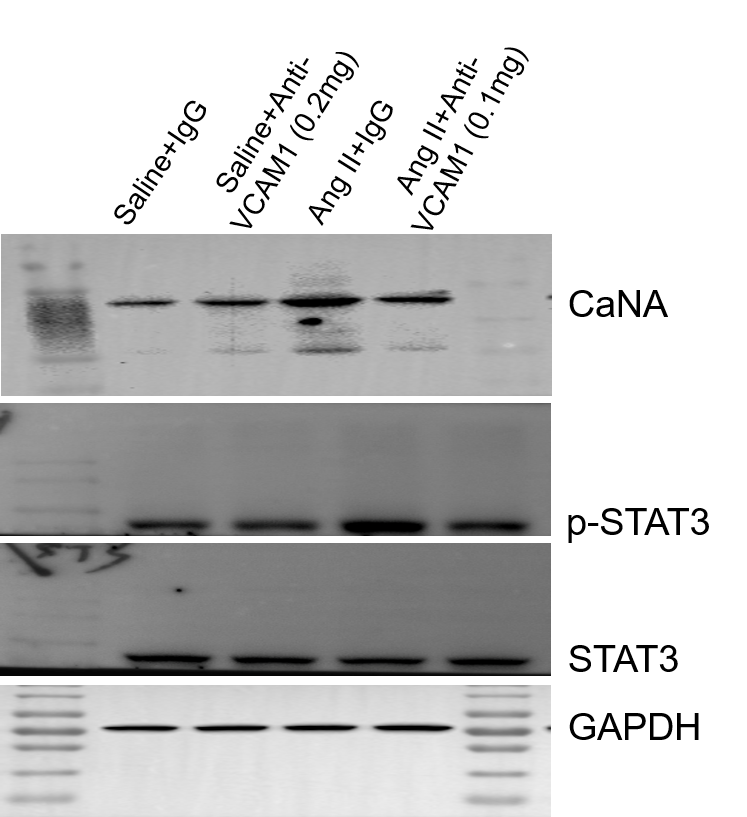


**Figure 6G** The mRNA levels of collagen I and collagen III in CFs

|  | ANF | BNP |
| --- | --- | --- |
| Saline+IgG | 0.91 | 1.26 |
|  | 1.47 | 1.05 |
|  | 0.70 | 0.74 |
|  | 1.23 | 0.80 |
|  | 1.05 | 0.91 |
|  | 0.64 | 1.25 |
|  | 0.91 | 1.26 |
| Saline+Anti-VCAM-1 | 1.46 | 0.72 |
|  | 1.25 | 0.98 |
|  | 1.56 | 0.91 |
|  | 0.45 | 1.03 |
|  | 0.60 | 1.14 |
|  | 0.63 | 0.92 |
| Ang II+IgG | 2.47 | 1.90 |
|  | 2.28 | 3.54 |
|  | 3.57 | 1.61 |
|  | 2.78 | 2.63 |
|  | 2.26 | 1.94 |
|  | 2.78 | 2.80 |
| Ang II+Anti-VCAM-1 | 1.27 | 1.14 |
|  | 1.62 | 0.94 |
|  | 1.30 | 1.11 |
|  | 1.53 | 1.18 |
|  | 1.12 | 1.47 |
|  | 0.87 | 1.03 |

**Figure 6H** Immunoblot of TGF-β1 and p-Smad2/3


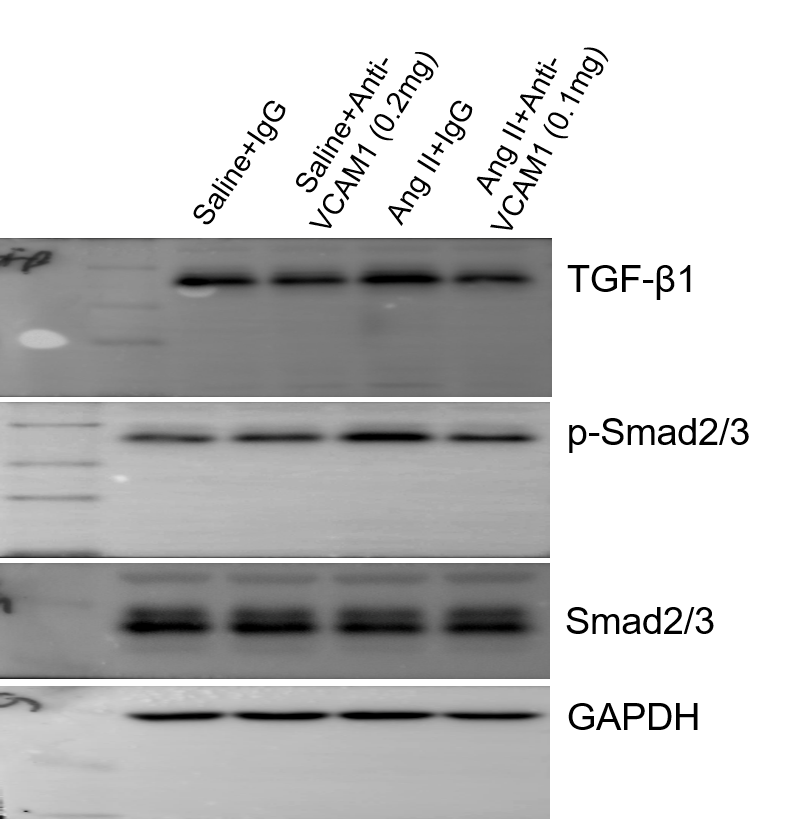

Supplement: Supplementary file 1 [file Table1.DOCX]
